# Supplementary material for: Normalization and variance stabilization of single-cell RNA-seq data using regularized negative binomial regression
Source: Genome Biol. 2019 Dec 23;20:296. doi: 10.1186/s13059-019-1874-1 (PMC6927181; doi:10.1186/s13059-019-1874-1)
Supplement: Supplementary file 1 — Additional file 1 Application of sctransform to additional UMI datasets. [file 13059_2019_1874_MOESM1_ESM.pdf]

Normalization and variance stabilization of single-cell RNA-seq data using regularized negative binomial regression

Additional file 1

Application of sctransform to additional UMI datasets

The following figures show results of our analysis and normalization method for five additional datasets:

1. Chromium Control (10X v2) [Svensson et al., 2017], ArrayExpress accession E-MTAB-5480, UMI count matrix available on `https://figshare.com/articles/svensson_chromium_control_h5ad/7860092`, sample 1
2. Human pancreas (CEL-seq2) GSE85241 [Muraro et al., 2016]
3. 10X Genomics 10k PBMCs from a Healthy Donor (v3 chemistry)
4. 10X Genomics 10k Brain Cells from an E18 Mouse (v3 chemistry)
5. 10X Genomics 10k Heart Cells from an E18 mouse (v3 chemistry)

10X Genomics datasets can be found at
`https://support.10xgenomics.com/single-cell-gene-expression/datasets/`

Figure legends

First figure (S1) – Overview of datasets and bias in log-normalized expression

Top Distribution of total UMI counts / cell ('sequencing depth').

Middle We placed genes into six groups, based on their average expression in the dataset.

Bottom For each gene group, we examined the average relationship between log-normalized expression and cell sequencing depth. We fit a smooth line for each gene individually and combined results based on the groupings. Black line shows mean, colored region indicates interquartile range. Values were scaled (z-scored) so that a single y-axis range could be used.

Second figure (S2) – Overview of bias in normalized expression

Top After normalizing data with scran [Lun et al., 2016a,b].

Bottom After normalizing data with regularized negative binomial regression models.

Remaining figures – Details of normalization and variance stabilization

A) Model parameters for genes detected in at least 0.1% of the cells for the NB regression model, plotted as a function of average gene abundance. The color of each point indicates a parameter uncertainty score as determined by bootstrapping (Methods). Pink line shows the regularized parameters obtained via kernel regression.

B) Standard deviation (σ) of NB regression model parameters across multiple bootstraps. Red points: σ for unconstrained NB model. Blue points: σ for regularized NB model, which is substantially reduced in comparison. Black trendline shows an increase in σ for low-abundance genes, highlighting the potential for overfitting in the absence of regularization.

C) Distribution of residual mean, across all genes, is centered at 0.

D) Density of residual gene variance peaks at 1, as would be expected when the majority of genes do not vary across cell types.

E) Variance of Pearson residuals is independent of gene abundance, demonstrating that the GLM has corrected for the mean-variance relationship inherent in the data. Genes with high residual variance (top 15 are labeled) tend to be cell-type markers. In contrast to a regularized NB, a Poisson error model does not fully capture the variance in highly expressed genes. An unconstrained (non-regularized) NB model overfits scRNA-seq data, attributing almost all variation to technical effects. As a result, even cell-type markers exhibit low residual variance. Mean-variance trendline shown in blue for each panel. In the case of Chromium Control even the highest residual variance for regularized NB is < 2.3. This highlights the effectiveness of our method as in the absence of biological variance no variable genes should remain after normalization.

References

A. T. Lun, K. Bach, and J. C. Marioni. Pooling across cells to normalize single-cell RNA sequencing data with many zero counts. *Genome Biology*, 17(1):1–14, 2016a. ISSN 1474760X. doi: 10.1186/s13059-016-0947-7. URL `http://dx.doi.org/10.1186/s13059-016-0947-7`.

A. T. Lun, D. J. McCarthy, and J. C. Marioni. A step-by-step workflow for low-level analysis of single-cell RNA-seq data with Bioconductor. *F1000Research*, 5:2122, 2016b. ISSN 2046-1402. doi: 10.12688/f1000research.9501.2. URL `https://f1000research.com/articles/5-2122/v2`.

M. J. Muraro, G. Dharmadhikari, D. Grün, N. Groen, T. Dielen, E. Jansen, L. van Gurp, M. A. Engelse, F. Carlotti, E. J. de Koning, and A. van Oudenaarden. A Single-Cell Transcriptome Atlas of the Human Pancreas. *Cell Systems*, 3(4):385–394.e3, oct 2016. ISSN 2405-4712. doi: 10.1016/j.cels.2016.09.002. URL `https://doi.org/10.1016/j.cels.2016.09.002`.

V. Svensson, K. N. Natarajan, L.-H. Ly, R. J. Miragaia, C. Labalette, I. C. Macaulay, A. Cvejic, and S. A. Teichmann. Power analysis of single-cell RNA-sequencing experiments. *Nature Methods*, 14:381, mar 2017. URL `https://doi.org/10.1038/nmeth.4220http://10.0.4.14/nmeth.4220https://www.nature.com/articles/nmeth.4220{\#}supplementary-information`.

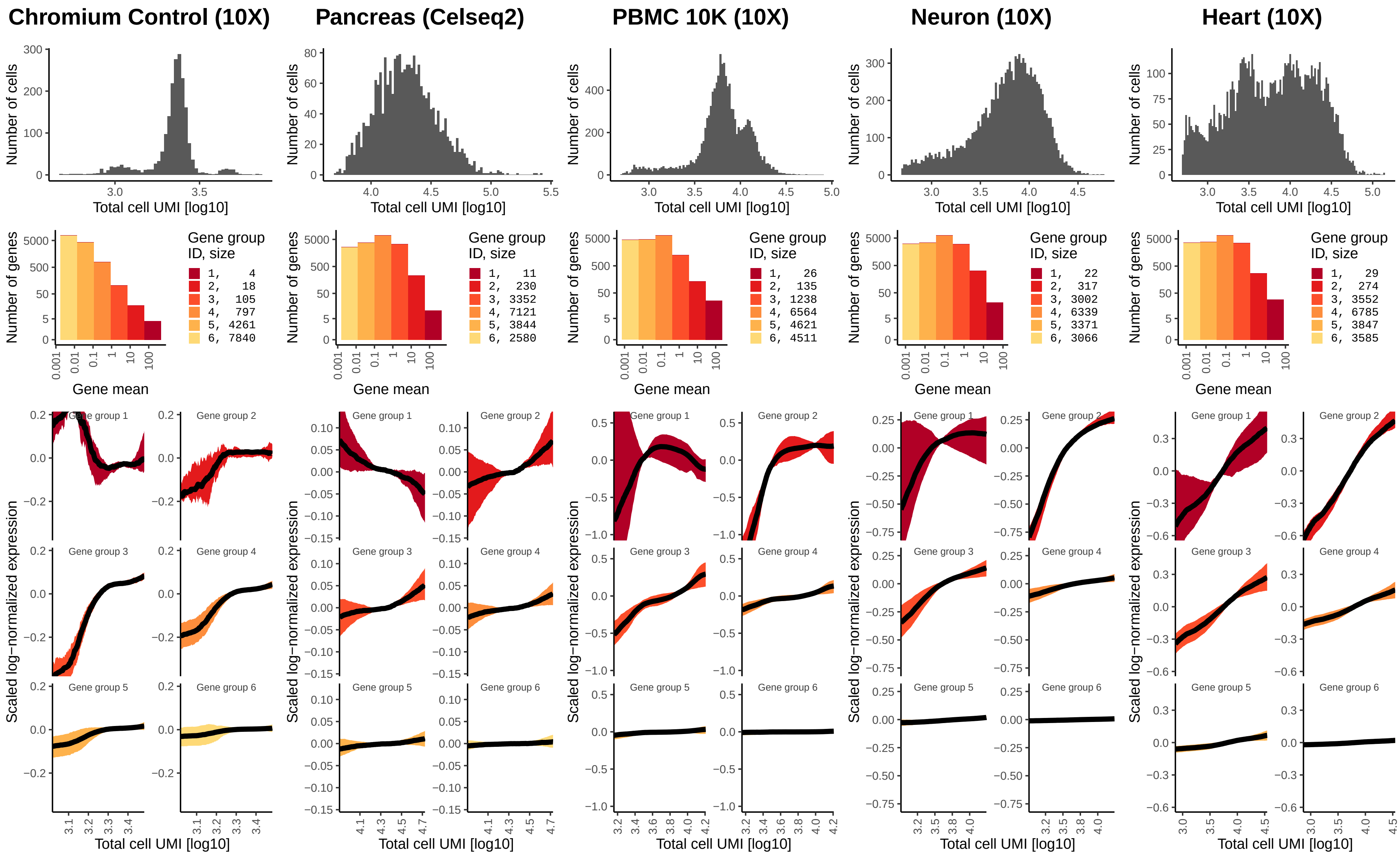
Chromium Control (10X)
Pancreas (Celseq2)
PBMC 10K (10X)
Neuron (10X)
Heart (10X)
Number of cells
Total cell UMI [log10]
Number of genes
Gene mean
Gene group ID, size
1, 4
2, 18
3, 105
4, 797
5, 4261
6, 7840
1, 11
2, 230
3, 3352
4, 7121
5, 3844
6, 2580
1, 26
2, 135
3, 1238
4, 6564
5, 4621
6, 4511
1, 22
2, 317
3, 3002
4, 6339
5, 3371
6, 3066
1, 29
2, 274
3, 3552
4, 6785
5, 3847
6, 3585
Gene group 1
Gene group 2
Gene group 3
Gene group 4
Gene group 5
Gene group 6
Scaled log−normalized expression
Total cell UMI [log10]

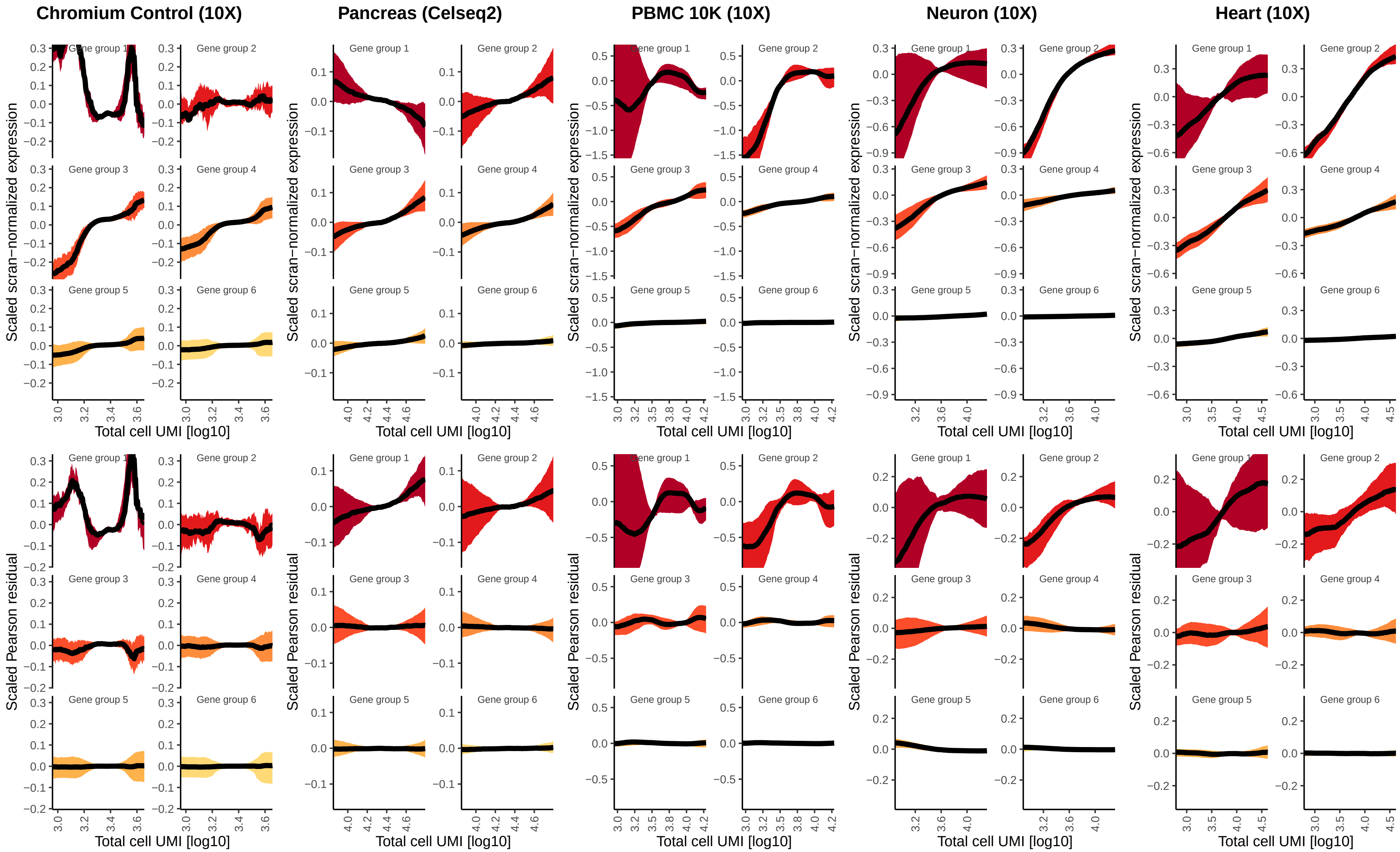

Chromium Control (10X)
Pancreas (Celseq2)
PBMC 10K (10X)
Neuron (10X)
Heart (10X)
Gene group 1
Gene group 2
Gene group 3
Gene group 4
Gene group 5
Gene group 6
Scaled scran−normalized expression
Scaled Pearson residual
Total cell UMI [log10]

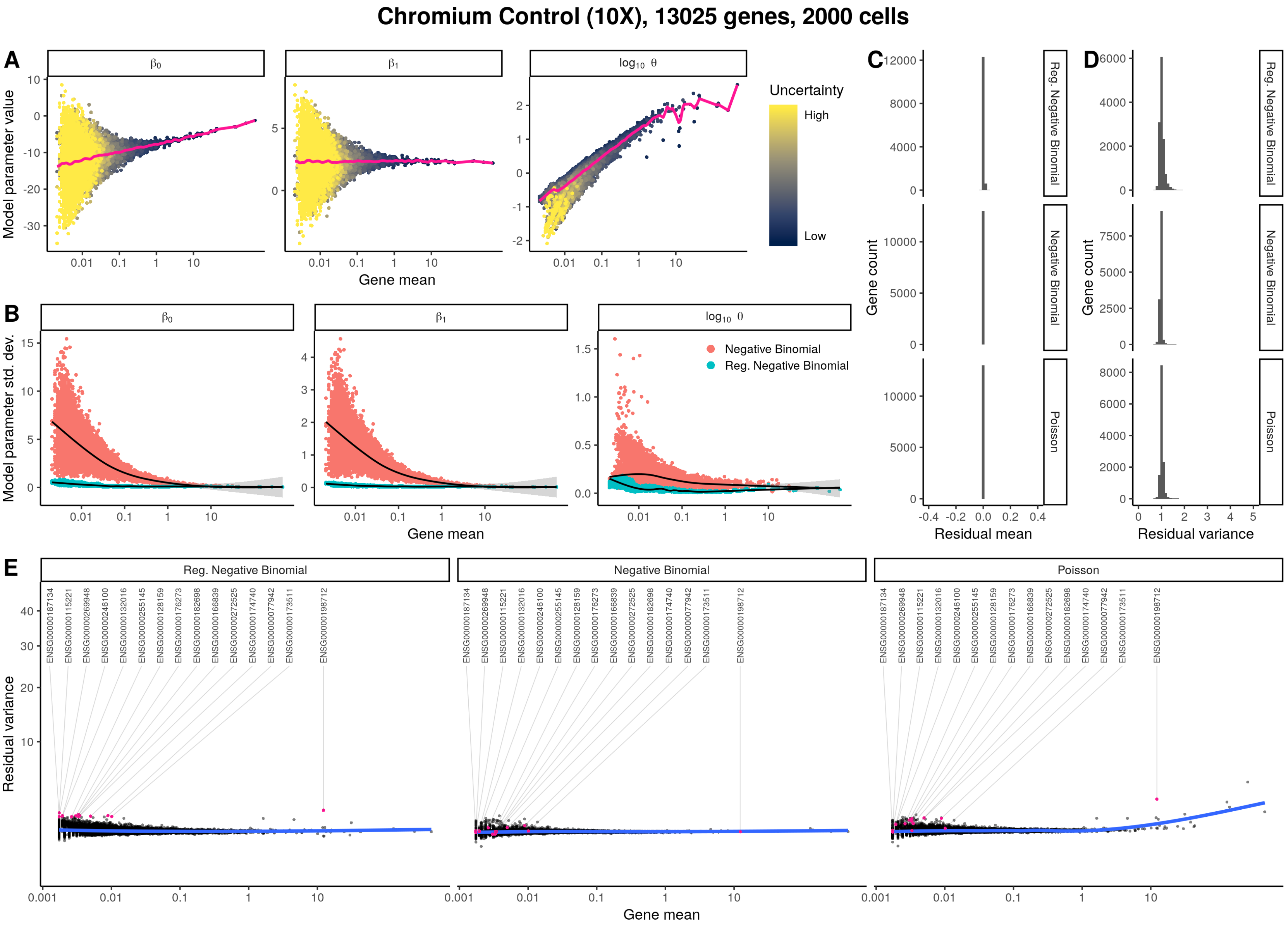
Chromium Control (10X), 13025 genes, 2000 cells
A
β0
β1
log10 θ
Model parameter value
Gene mean
Uncertainty
High
Low
B
Model parameter std. dev.
Negative Binomial
Reg. Negative Binomial
C
Gene count
Residual mean
Reg. Negative Binomial
Negative Binomial
Poisson
D
Residual variance
E
Residual variance
ENSG00000187134
ENSG00000115221
ENSG00000269948
ENSG00000246100
ENSG00000132016
ENSG00000255145
ENSG00000128159
ENSG00000176273
ENSG00000182698
ENSG00000166839
ENSG00000272525
ENSG00000174740
ENSG00000077942
ENSG00000173511
ENSG00000198712

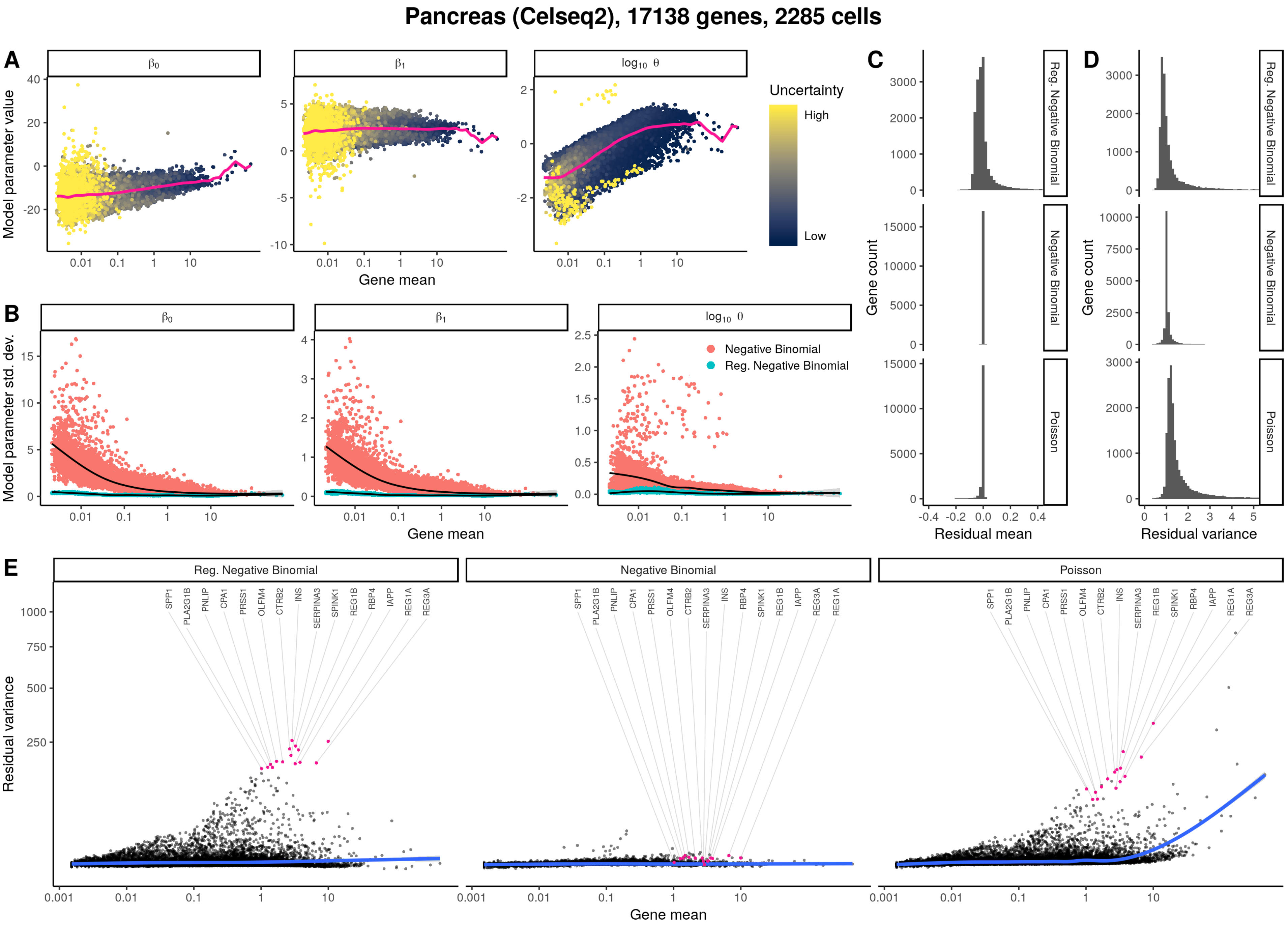

Pancreas (Celseq2), 17138 genes, 2285 cells
A
B
C
D
E
Model parameter value
Gene mean
Uncertainty
High
Low
Model parameter std. dev.
Negative Binomial
Reg. Negative Binomial
Gene count
Residual mean
Residual variance
Poisson
Residual variance
SPP1
PLA2G1B
PNLIP
CPA1
PRSS1
OLFM4
CTRB2
INS
SERPINA3
SPINK1
REG1B
RBP4
IAPP
REG1A
REG3A

PBMC (10X), 17095 genes, 11769 cells

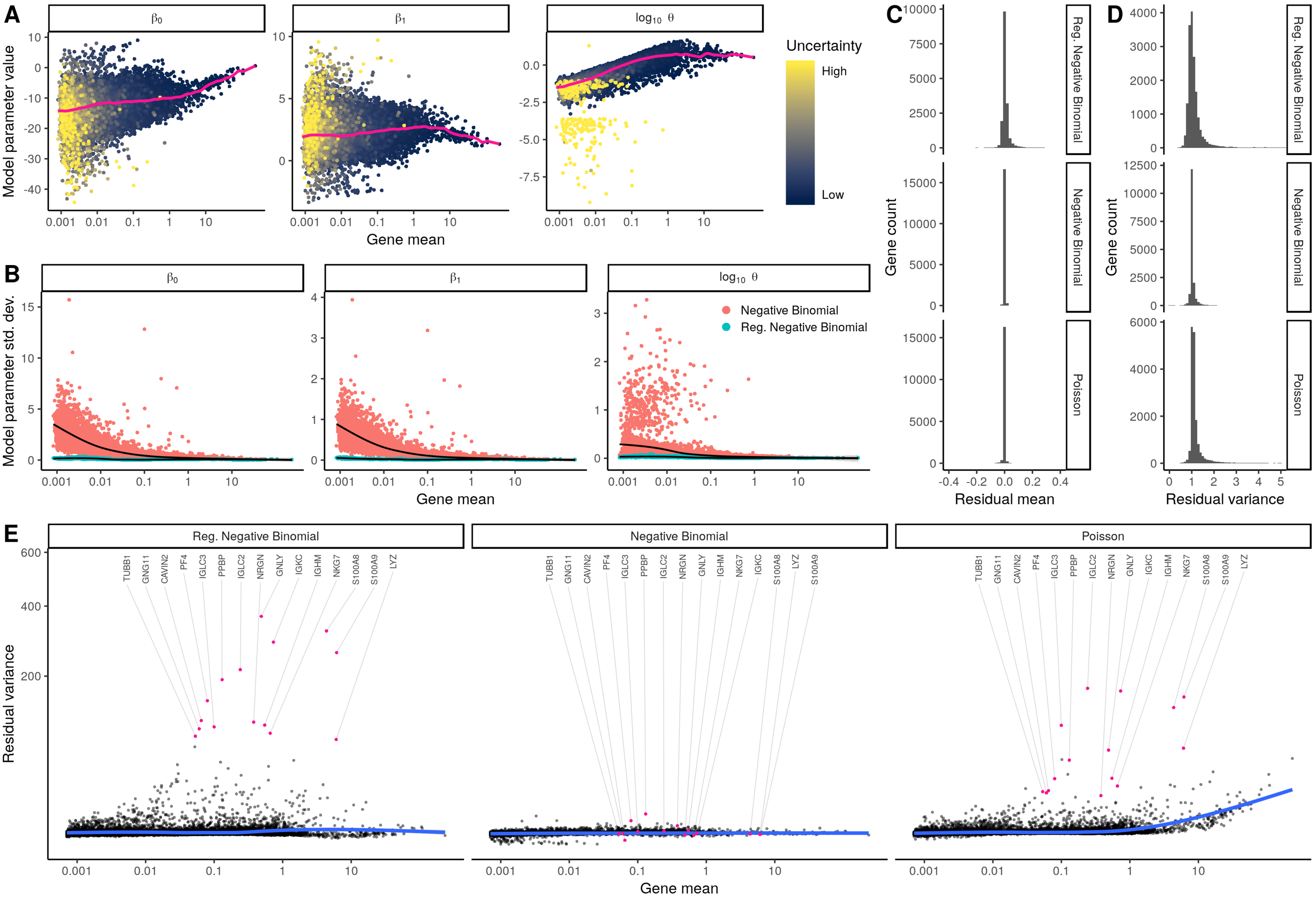

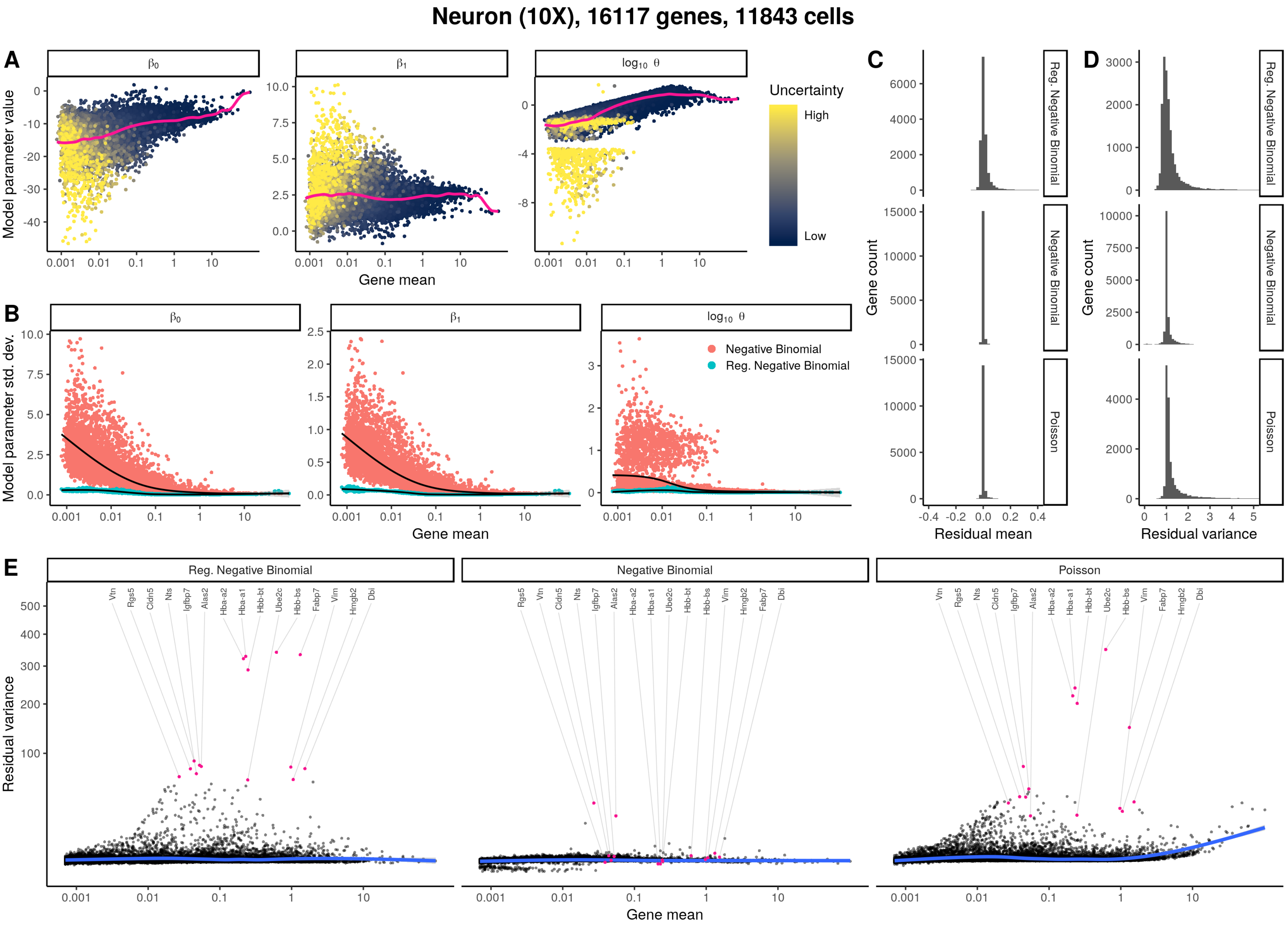

Neuron (10X), 16117 genes, 11843 cells
A
B
C
D
E
Model parameter value
Gene mean
Uncertainty
High
Low
Model parameter std. dev.
Negative Binomial
Reg. Negative Binomial
Poisson
Gene count
Residual mean
Residual variance

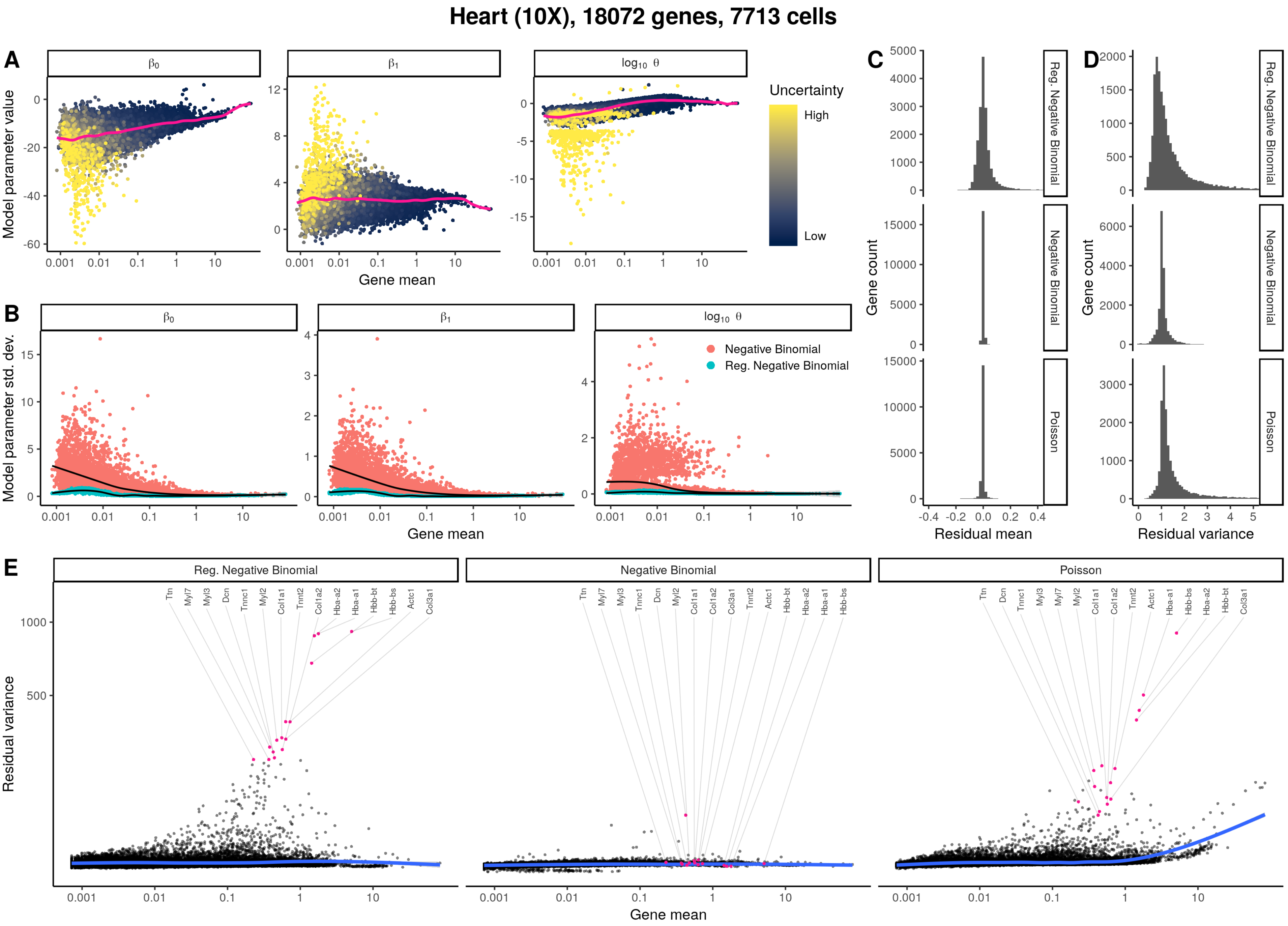
Heart (10X), 18072 genes, 7713 cells
A
B
C
D
E
Model parameter value
Model parameter std. dev.
Gene mean
Uncertainty
High
Low
Negative Binomial
Reg. Negative Binomial
Poisson
Gene count
Residual mean
Residual variance
