# Supplementary material for: Normalization and variance stabilization of single-cell RNA-seq data using regularized negative binomial regression
Source: Genome Biol. 2019 Dec 23;20:296. doi: 10.1186/s13059-019-1874-1 (PMC6927181; doi:10.1186/s13059-019-1874-1)
Supplement: Supplementary file 2 — Additional file 2 Supplementary figures. [file 13059_2019_1874_MOESM2_ESM.pdf]

Normalization and variance stabilization of single-cell RNA-seq data using regularized negative binomial regression

Additional file 2

Supplementary figures

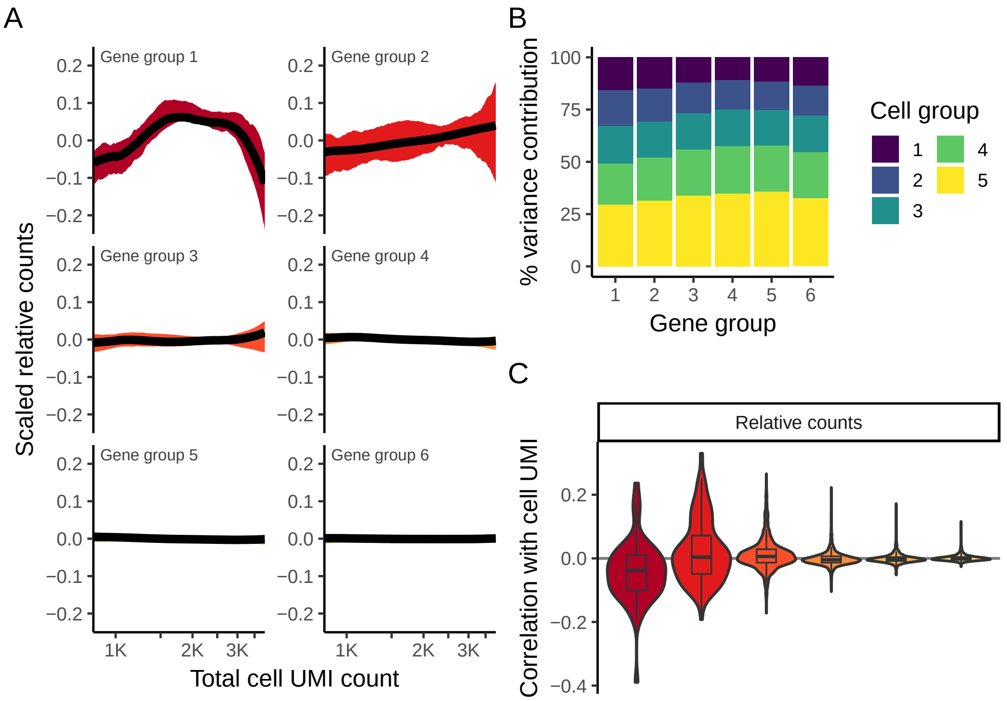

Fig. S1: ’Relative counts’ normalization of 33K PBMC data set. **A,B)** Visualizations are analogous to Fig. 1D,E, but calculated using ’relative counts’ normalization. **C)** Boxplot of Pearson correlations between ’relative counts’ and total cell UMI counts for each of the six gene bins.

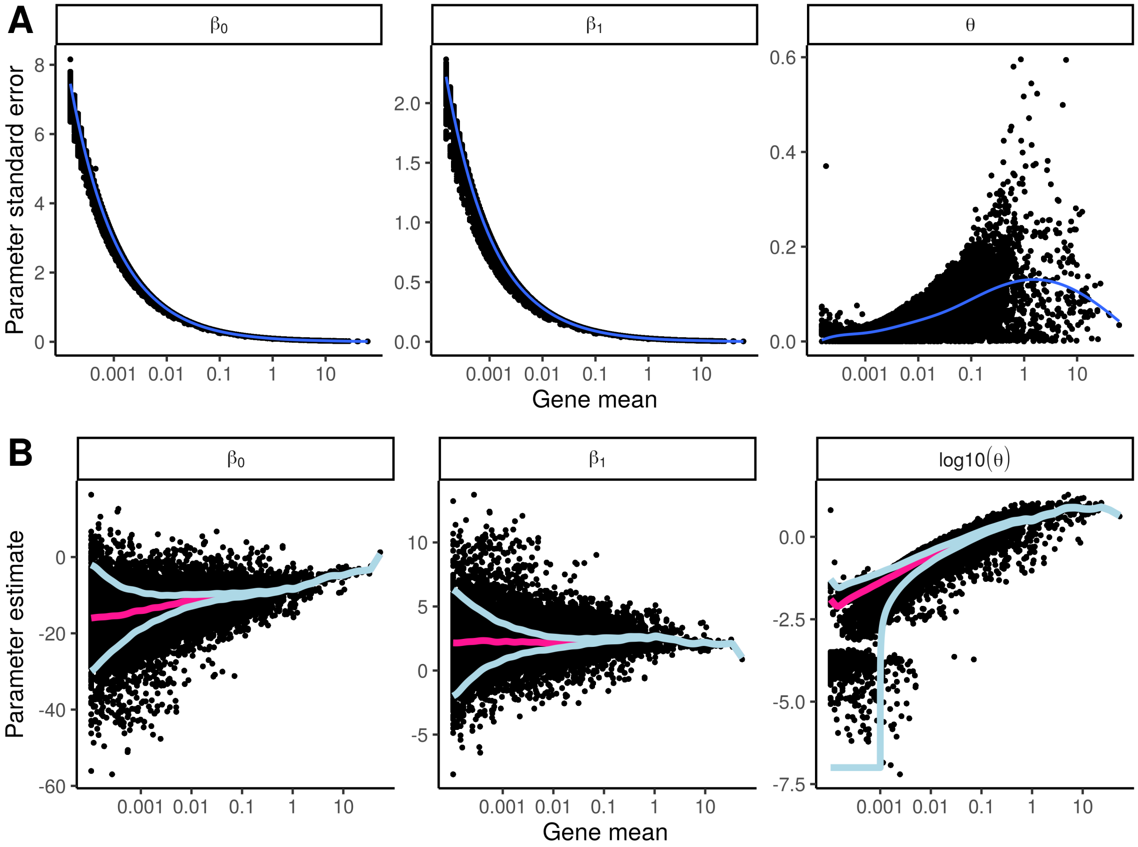

Fig. S2: Parameter estimates for unregularized negative binomial regression exhibit high uncertainty, particularly for lowly abundant genes. Related to the same dataset as Fig. 2, but using the Fisher information matrix to estimate uncertainty as an alternative to bootstrapping. **A)** Standard error as a function of gene abundance for all three parameters. Trendline shown in blue. **B)** Per-gene estimates for all three parameters. Light blue line indicates the 95% confidence interval trendline. Pink line (regularized parameter estimates) and data points are the same as in Fig. 2A

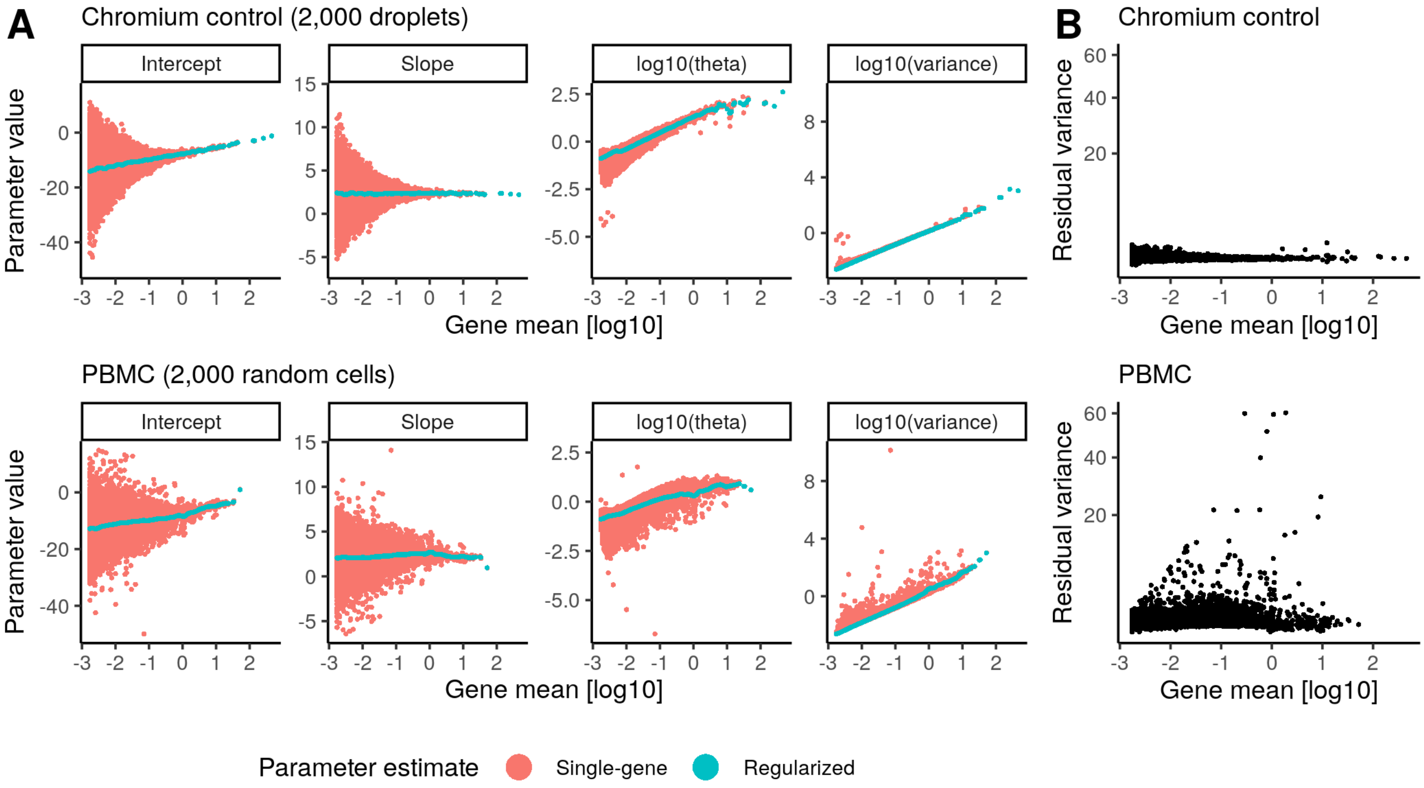

Fig. S3: Performing regularized negative binomial regression on a 'Chromium control' dataset (Svensson et al. [2017]), where each droplet represents a technical replicate of the same human brain bulk RNA pool. **A)** Top row: Per-gene and regularized parameter estimates for this dataset. Even in the absence of biological variance, there is substantial variation in the estimation of single-gene parameters due to overfitting. Bottom row: For comparison, estimates for the PBMC dataset, randomly downsampled to 2,000 cells. **B)** Per-gene variance of pearson residuals after applying negative binomial regression. In the control dataset (top), we correctly do not identify genes with high residual variance.

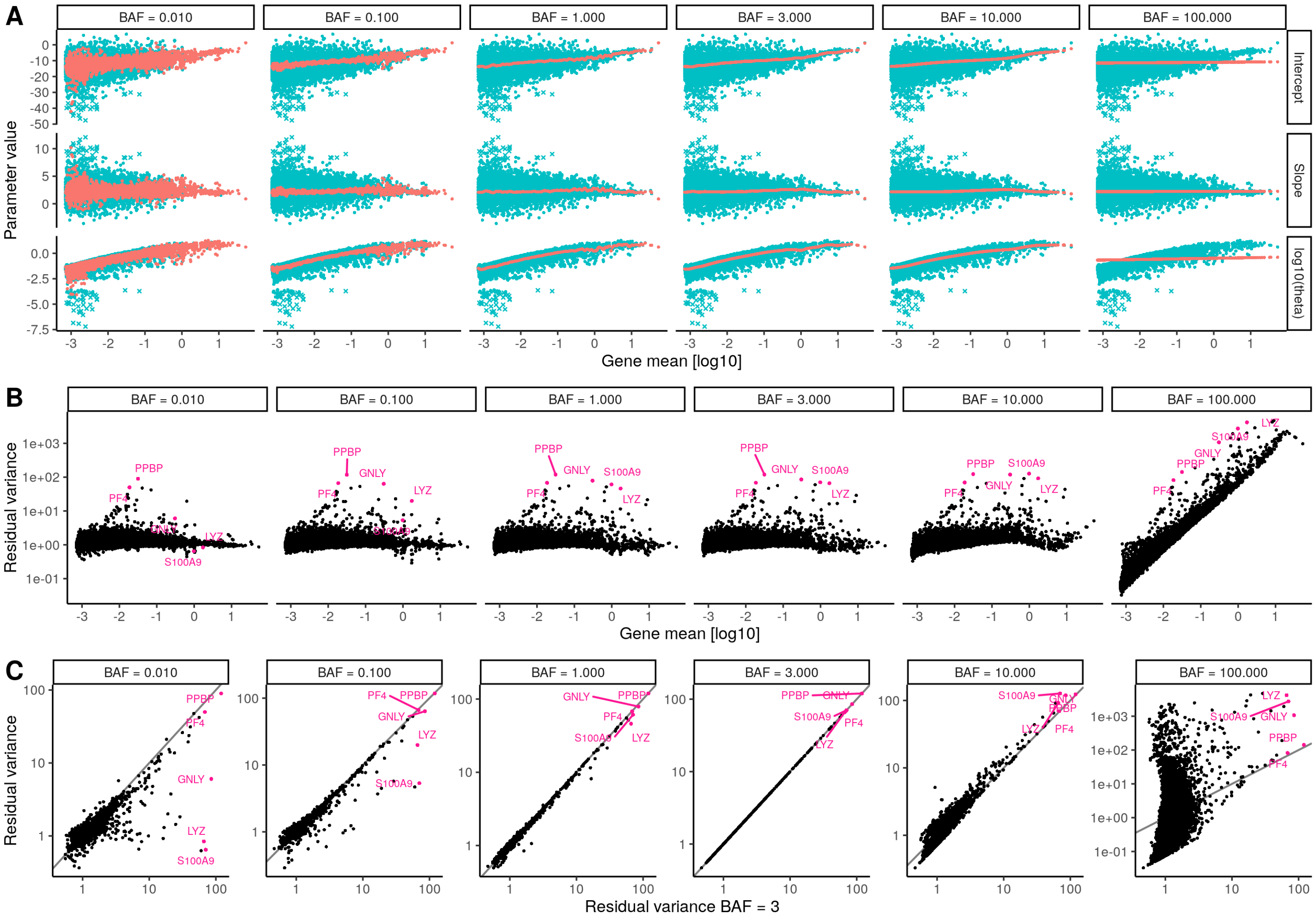

Fig. S4: Bandwidth adjustment factor (BAF) sensitivity analysis. **A)** Regularized parameter estimates (red line) for five different values of BAF. Blue points represent single-gene parameter estimates, and are the same in all panels. **B)** Gene mean versus residual variance relationship for different BAF values. Setting BAF too high fails to correct for the mean/variance relationship in the data. **C)** Per-gene residual variance for different BAF, compared to the default value (BAF=3). Results for all panels are robust within a range of 1 to 10.

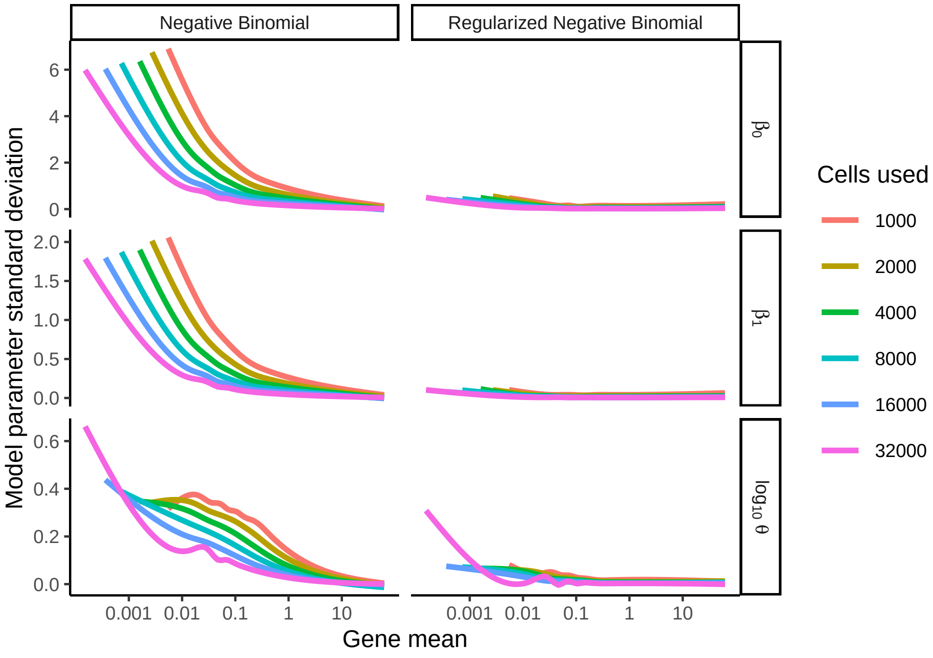

Fig. S5: Trendlines showing the relationship between sample size (number of cells) and model parameter uncertainty. Parameter uncertainty decreases for more highly abundant genes, or larger dataset sizes. Regularization substantially reduces uncertainty independent of sample size.

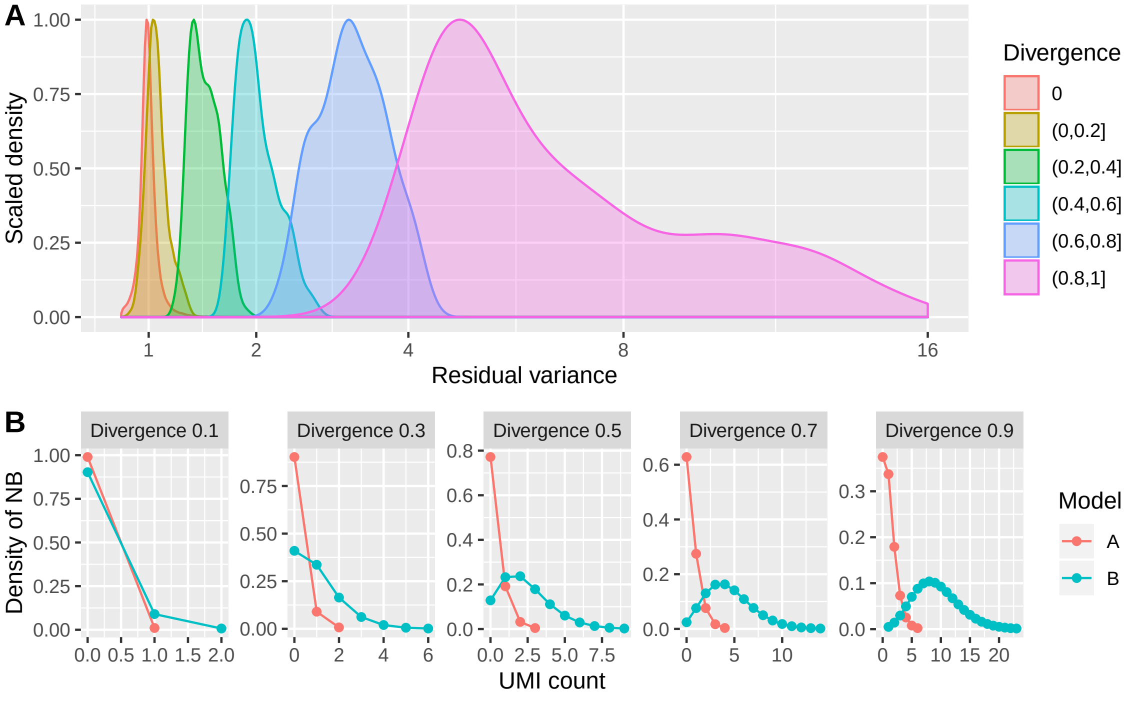

Fig. S6: We simulated data (see Methods) to assess sensitivity of variable gene detection of our method. We used a balanced mixture to introduce a 10-fold change in mean UMI count for 10% randomly selected genes. **A)** Residual variances for genes grouped by Jensen-Shannon divergence of the generating models. A divergence of 0 indicates genes that were generated from one model (non-variable), and the residual variance of these genes is tightly centered around 1. Except for genes in the lowest divergence bin (which have exceptionally low detection rate), we can successfully use residual variance to distinguish variable genes in the dataset, suggesting that our regularized regression procedure is effectively modeling these data. **B)** Representative examples of models with different divergence. The model parameters were extracted from the regularized models used to generate the data in A.

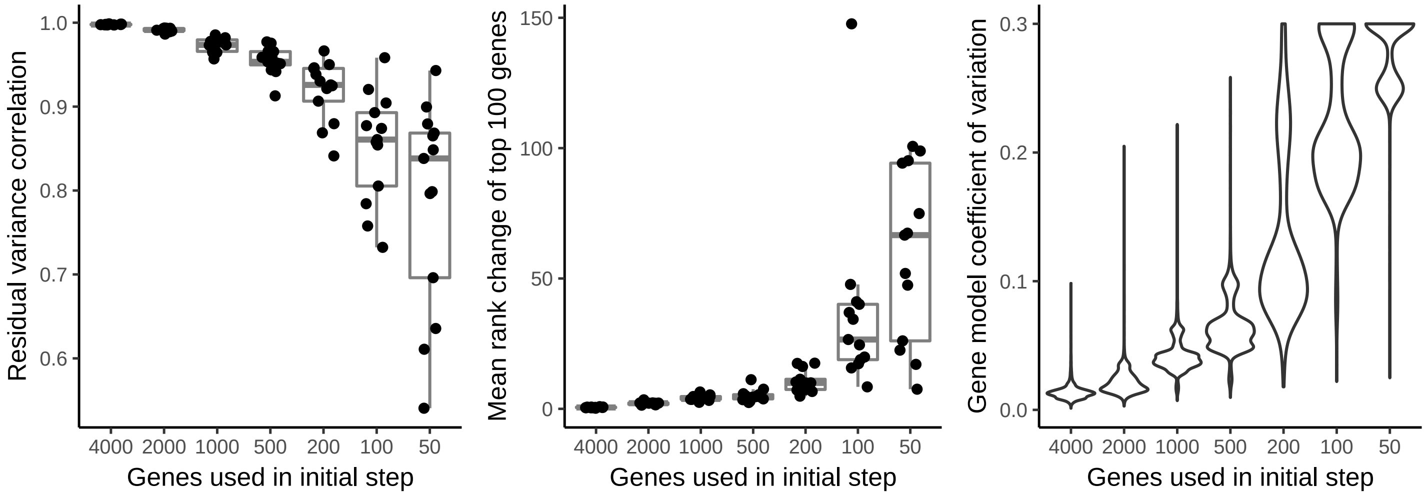

Fig. S7: A representative set of 2,000 genes is sufficient for learning regularized models. **A,B)** Comparing models learned using only a subset of genes and models learned using all 16,809 genes. **A)** Pearson correlation of gene residuals **B)** Mean rank change of top 100 variable genes as determined by residual variance. **C)** Coefficient of variation of sum of squared residuals across multiple samples; All panels show results of 13 random samples per gene subset size

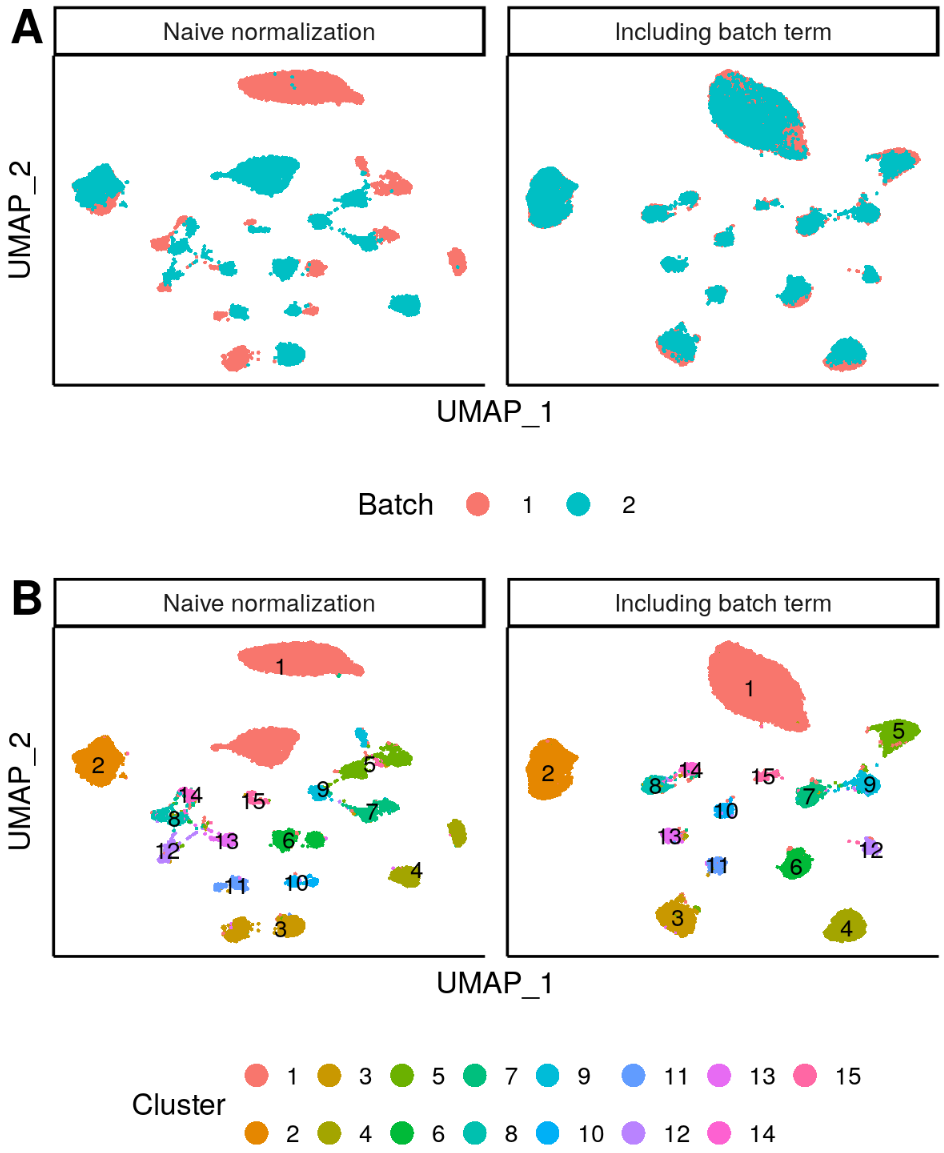

Fig. S8: Batch-correction during normalization. **A)** UMAP embedding of the bipolar cell dataset before and after including a batch term during normalization. When applying `sctransform` without the batch indicator variable (i.e. batch-naive normalization), we see clear separation per batch, but when including a batch term in the regression model used for normalization, the batches align. **B)** Same as above, but colors indicate clusters of the original study. We include this example as a demonstration for how additional nuisance parameters can be included in the GLM framework, but note that when cell-type specific batch effects or present, or there is a shift in the percentage of cell types across experiments, non-linear batch effect correction strategies are needed (Stuart et al. [2019])

References

T. Stuart, A. Butler, P. Hoffman, C. Hafemeister, E. Papalexi, W. M. Mauck III, Y. Hao, M. Stoeckius, P. Smibert, and R. Satija. Comprehensive Integration of Single-Cell Data. *Cell*, 177(7):1888–1902.e21, jun 2019. ISSN 0092-8674. doi: 10.1016/j.cell.2019.05.031. URL https://doi.org/10.1016/j.cell.2019.05.031.

V. Svensson, K. N. Natarajan, L.-H. Ly, R. J. Miragaia, C. Labalette, I. C. Macaulay, A. Cvejic, and S. A. Teichmann. Power analysis of single-cell RNA-sequencing experiments. *Nature Methods*, 14:381, mar 2017. URL https://doi.org/10.1038/nmeth.4220http://10.0.4.14/nmeth.4220https://www.nature.com/articles/nmeth.4220{#}supplementary-information.
