# Supplementary material for: Normalization and variance stabilization of single-cell RNA-seq data using regularized negative binomial regression
Source: Genome Biol. 2019 Dec 23;20:296. doi: 10.1186/s13059-019-1874-1 (PMC6927181; doi:10.1186/s13059-019-1874-1)

Normalization and variance stabilization of single-cell RNA-seq data using regularized negative binomial regression

Additional file 3: Using sctransform in Seurat

Christoph Hafemeister & Rahul Satija

2019-10-29

This vignette shows how to use the sctransform wrapper in Seurat.

Install sctransform and Seurat from CRAN

```
install.packages("sctransform")
install.packages("Seurat")
library(Seurat)
library(sctransform)
```

Load data and create Seurat object

```
pbmc_data <- Read10X(data.dir = "~/Downloads/pbmc3k_filtered_gene_bc_matrices/hg19/")
pbmc <- CreateSeuratObject(counts = pbmc_data)
```

For reference, we first apply the standard Seurat workflow, with log-normalization

```
pbmc_logtransform <- pbmc
pbmc_logtransform <- NormalizeData(pbmc_logtransform, verbose = FALSE)
pbmc_logtransform <- FindVariableFeatures(pbmc_logtransform, verbose = FALSE)
pbmc_logtransform <- ScaleData(pbmc_logtransform, verbose = FALSE)
pbmc_logtransform <- RunPCA(pbmc_logtransform, verbose = FALSE)
pbmc_logtransform <- RunUMAP(pbmc_logtransform, dims = 1:20, verbose = FALSE)
```

For comparison, we now apply sctransform normalization

```
# Note that this single command replaces NormalizeData, ScaleData, and
# FindVariableFeatures.  Transformed data will be available in the SCT
# assay, which is set as the default after running sctransform
pbmc <- SCTransform(object = pbmc, verbose = FALSE)
```

Perform dimensionality reduction by PCA and UMAP embedding

```
# These are now standard steps in the Seurat workflow for visualization and
# clustering
pbmc <- RunPCA(object = pbmc, verbose = FALSE)
pbmc <- RunUMAP(object = pbmc, dims = 1:20, verbose = FALSE)

pbmc <- FindNeighbors(object = pbmc, dims = 1:20, verbose = FALSE)
pbmc <- FindClusters(object = pbmc, verbose = FALSE)
```

Visualize the clustering results on the sctransform and log-normalized embeddings.

```
pbmc_logtransform$clusterID <- Idents(pbmc)
Idents(pbmc_logtransform) <- "clusterID"
plot1 <- DimPlot(object = pbmc, label = TRUE) + NoLegend() + ggtitle("sctransform")
plot2 <- DimPlot(object = pbmc_logtransform, label = TRUE) + NoLegend() + ggtitle("Log-normalization")
CombinePlots(list(plot1, plot2))
```

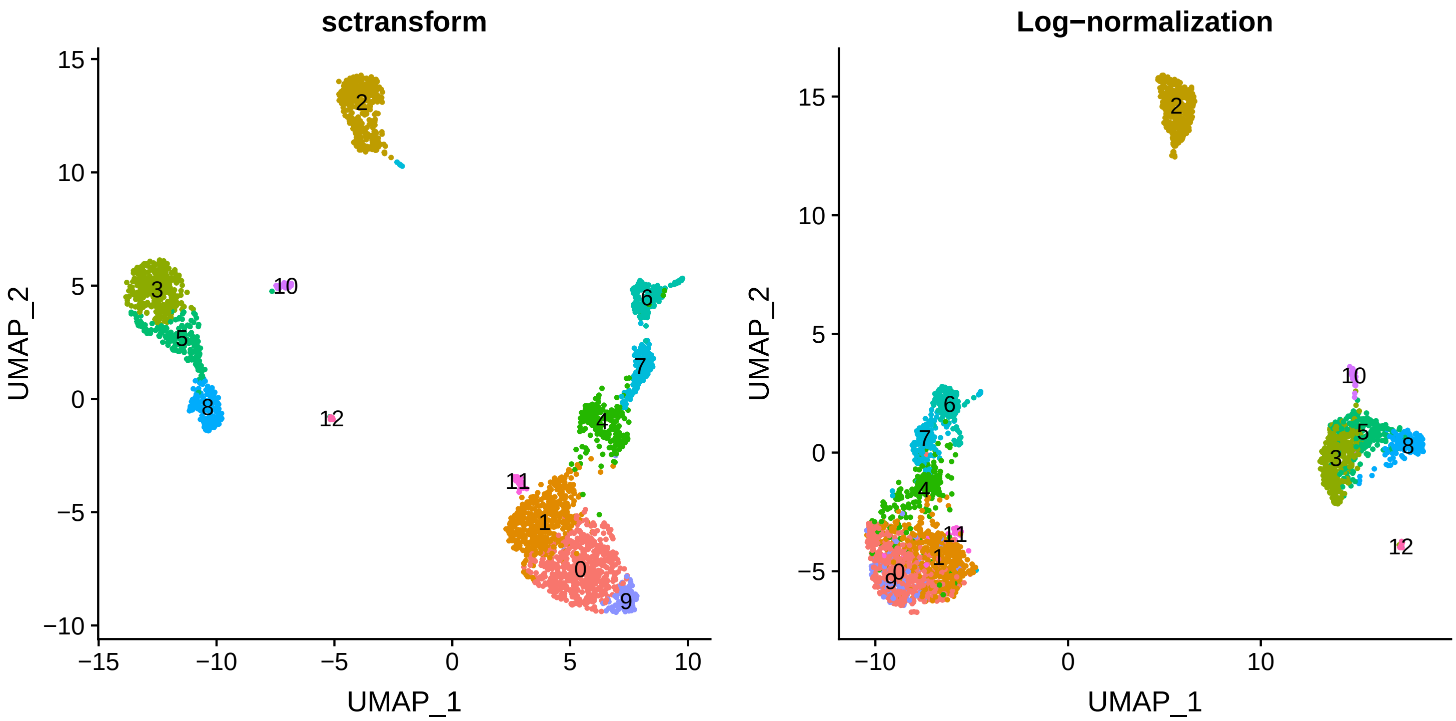

Users can individually annotate clusters based on canonical markers. However, the sctransform normalization reveals sharper biological distinctions compared to the log-normalized analysis. For example, note how clusters 0, 1, 4, 9, and 11 (all distinct clusters of T cells), are blended together in log-normalized analyses. The sctransform analysis reveals:

- Clear separation of three CD8 T cell clusters (naive, memory, effector), based on CD8A, GZMK, CCL5, GZMK expression
- Clear separation of three CD4 T cell clusters (naive, memory, IFN-activated) based on S100A4, CCR7, IL32, and ISG15
- Additional developmental sub-structure in B cell cluster, based on TCL1A, FCER2
- Additional sub-structure within NK cell cluster (CD56dim vs. bright), based on XCL1 and FCGR3A

```
# Visualize canonical marker genes as violin plots.
VlnPlot(object = pbmc, features = c("CD8A", "GZMK", "CCL5", "S100A4", "S100A4",
    "CCR7", "ISG15", "CD3D"), pt.size = 0.2, ncol = 4)
```

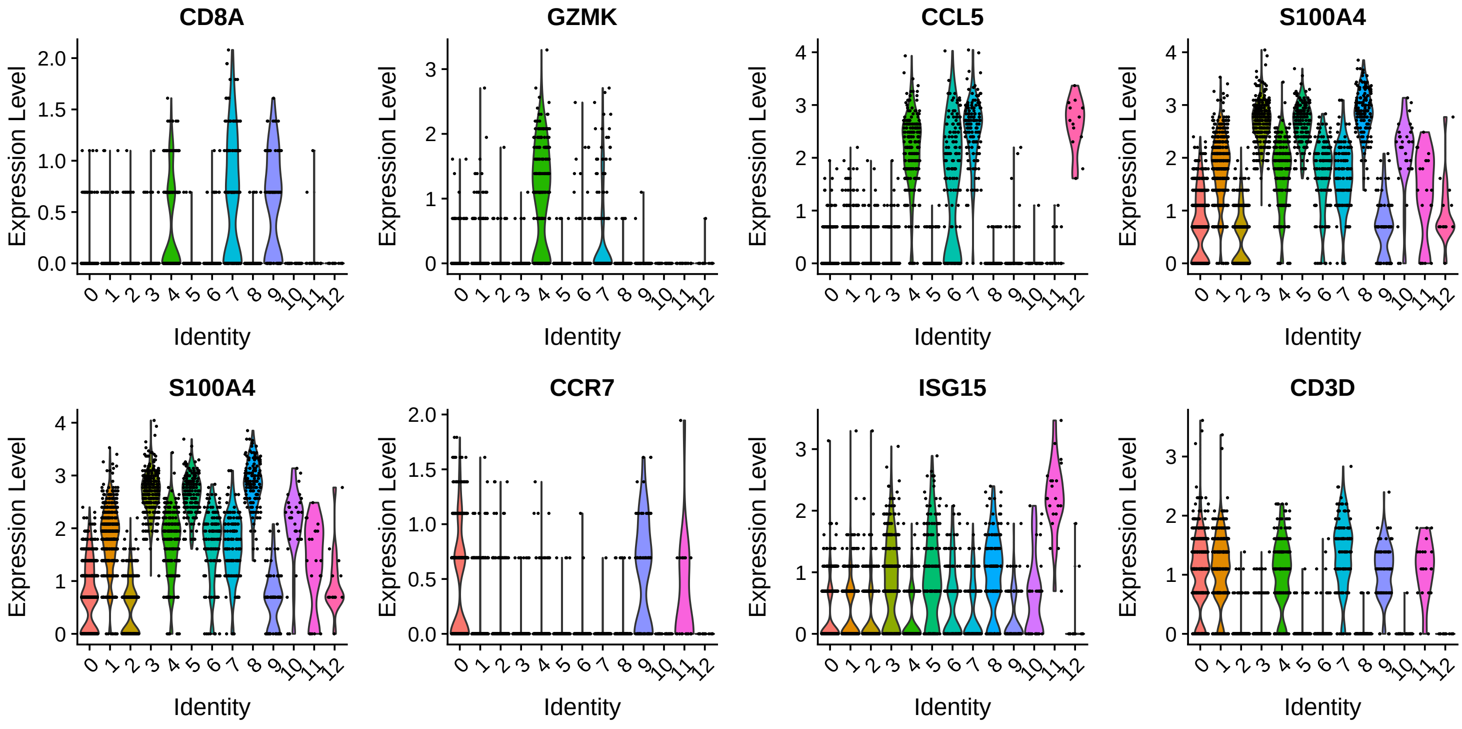


```
# Visualize canonical marker genes on the sctransform embedding.
FeaturePlot(object = pbmc, features = c("CD8A", "GZMK", "CCL5", "S100A4", "S100A4",
```

```
    "CCR7"), pt.size = 0.2, ncol = 3)
```

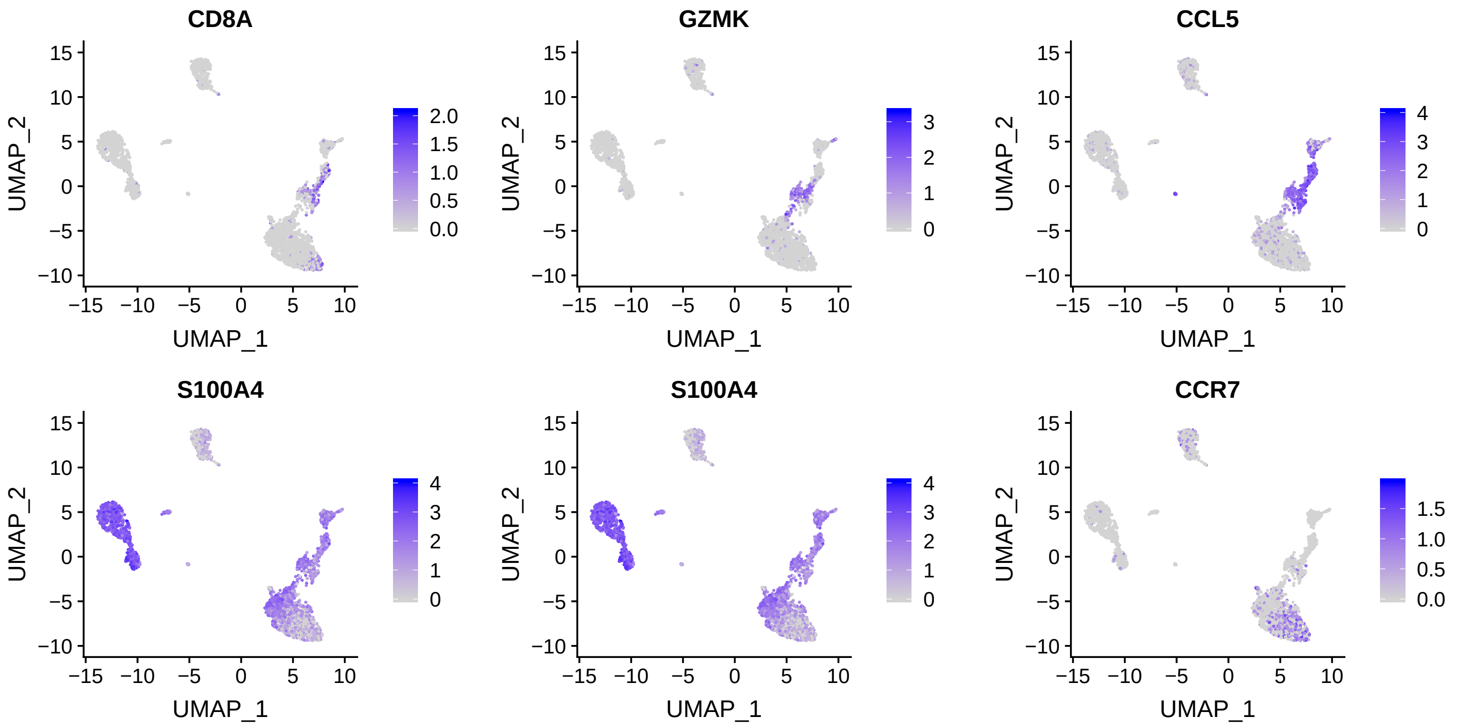


```
FeaturePlot(object = pbmc, features = c("CD3D", "ISG15", "TCL1A", "FCER2", "XCL1",
    "FCGR3A"), pt.size = 0.2, ncol = 3)
```

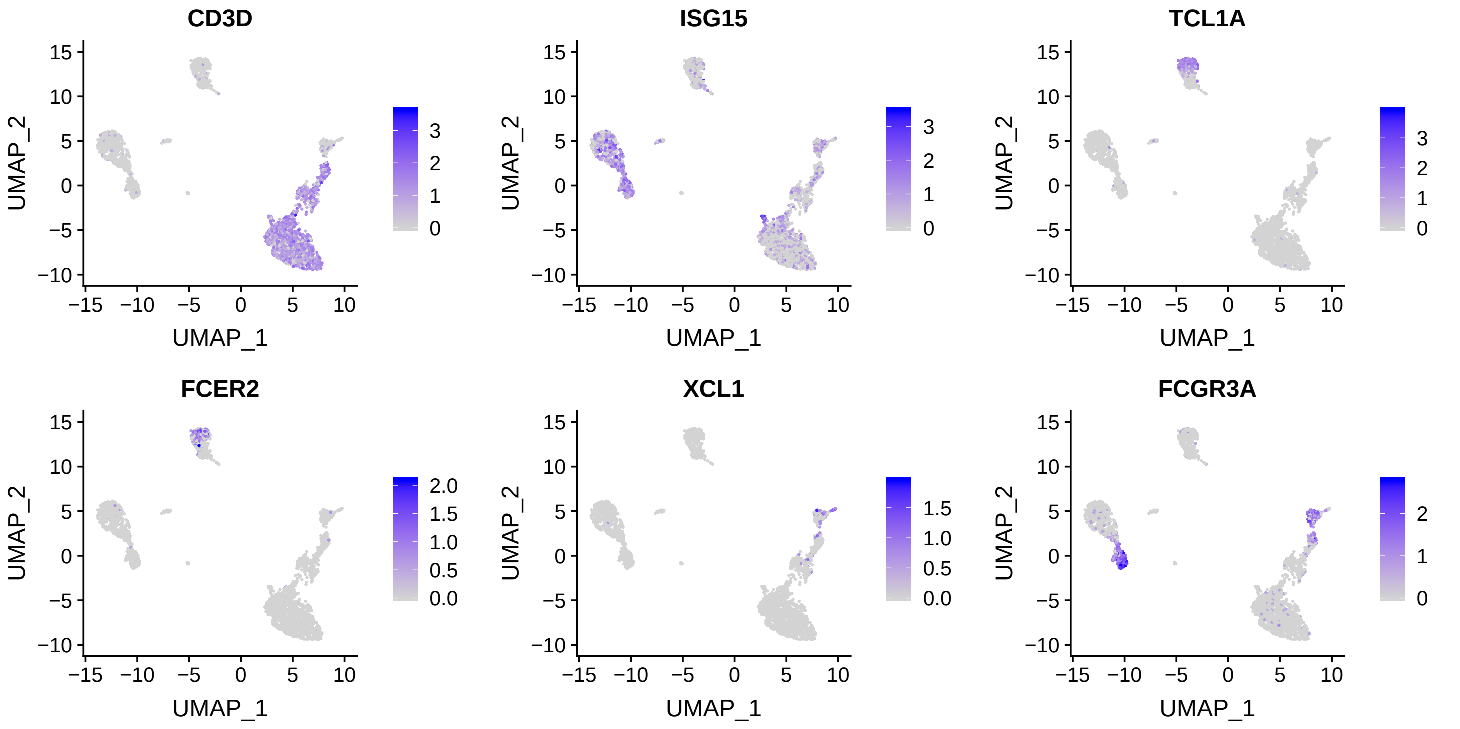

Supplement: Supplementary file 3 — Additional file 3 Guide to using sctransform in seurat. [file 13059_2019_1874_MOESM3_ESM.pdf]
