# Supplementary material for: Normalization and variance stabilization of single-cell RNA-seq data using regularized negative binomial regression
Source: Genome Biol. 2019 Dec 23;20:296. doi: 10.1186/s13059-019-1874-1 (PMC6927181; doi:10.1186/s13059-019-1874-1)
Supplement: Supplementary file 4 — Additional file 4 Review history. [file 13059_2019_1874_MOESM4_ESM.docx]

Review History

**First round of review**

**Reviewer 1**

**Were you able to assess all statistics in the manuscript, including the appropriateness of statistical tests used?**

**Yes: All statistics are valid and appropriate.**

**Were you able to directly test the methods?**

Yes

**Comments to author:**

In their present manuscript Hafemeister and Satija identify technical variability in single-cell RNA-sequencing data associated with variable total transcript counts across cells as a major confounding factor for the analysis of such data. By stratifying genes into groups of similar abundance they demonstrate that commonly used normalization schemes applying the same size factor to all genes cannot eliminate such kind of variability.

To solve this problem for UMI-based count data they propose a gene-specific normalization scheme, utilizing a general linear model with a negative binomial link function, the total UMI count per cell as explanatory variable and the UMI count vector for a given gene as response variable. Since per-gene regression appears to overfit the data, which they conclude from the instability of the parameter estimates during bootstrapping, they propose a regularization scheme. In this approach kernel regression is applied to learn the global trend from genes with similar abundance. The Pearson residuals obtained from these regularized parameters are then used as normalized gene expression estimates.

They show that these residuals are indeed not affected by sequencing-depth associated technical noise anymore. Importantly, they demonstrate that, while unconstrained negative binomial regression eliminates biological variability, a Poisson regression is insufficient to remove overdispersed technical noise - in agreement with previous studies.

They finally show that their normalization and variance stabilization corrects for sequencing-depth induced batch effects and substantially improves differential gene expression analysis in the presence of such nuisance factors.

The study is technically very well executed and the authors show convincing data demonstrating the power of their approach. The method represents a novel strategy to derive gene-dependent size factors and will be very useful for the community to discover more structure in single-cell datasets but also as a basis for future methods development.

However, I have a few points that need to be addressed:

1)	I like the idea of regularizing the regression parameter. The authors make the point that the parameter instability during bootstrapping is explained by systematic biological variability in the data, which could be different for any subsample of cells. Although this makes intuitive sense, this hypothesis should be directly tested, e.g., by analyzing pool-and-split RNA-samples without any biological variability. For instance, the authors could use the pool-and-split data from Grün et al., 2014, Nature Methods. It would be reassuring if the inferred parameters were more stable for these data.

2)	Another concern is the uncertainty of the regularization for highly expressed genes. This is particularly apparent for the pancreas and the heart dataset in supplementary note 1. The kernel regression for these few highly expressed genes should perhaps incorporate more of the trend from the more lowly expressed genes to obtain a smoother dependency. It is in general not clear to me how the authors motivate their choice for the bandwidth of the kernel regression. This parameter should perhaps depend on the distribution data points (gene density as a function mean expression) which might be different for every dataset.

3)	It was demonstrated in the manuscript that the normalization method successfully eliminates batch effects associated with variability in sequencing depth and improves the identification of differentially expressed genes in the presence of this technical noise. However, the authors did not sufficiently test in a quantitative manner how their approach affects cell type identification by clustering. The PBMC dataset shown in Figure 6A is not very challenging, due to the limited number of cell types and the clear separation between them. It would be useful to compare clustering after using their method versus log-normalization and other normalization schemes (e.g. scran) for datasets with more cell type variability, ideally comprising sub-types that are more similar to each other. For example, they could analyse the intestinal data from Haber et al., Nature , 2017, and, in particular, test if they can recapitulate the taxonomy of enteroendocrine cells in Figure 3. This benchmarking should be done in a quantitative way, e.g. ,by computing the Rand index (against a curated annotation from the original manuscript) and the silhouette coefficients.

4)	Along the same lines the authors should test the effect of their normalization method on trajectory inference. In general, trajectory inference is a challenging task which requires the recovery of continuous manifolds in the data. It would be important to know if the proposed normalization method disrupts continuous trajectories. The authors could apply established methods for trajectory inference (PAGA, diffusion maps, Monocle3 etc.) on systems with well-established lineage information such as the bone marrow after different ways of normalization. Data from Dahlin et al., Blood, 2018 would be an option.

**Reviewer 2**

**Were you able to assess all statistics in the manuscript, including the appropriateness of statistical tests used?**

**Yes: Method paper, analysis code provided**

**Were you able to directly test the methods?**

No

**Comments to author:**

\##

Review of Normalization and variance stabilization of single-cell RNA-seq data using regularized negative binomial regression

Summary:

The authors set out to establish a normalisation technique for scRNA-seq data that takes the count noise and total number of UMIs into account. They concentrate their efforts on defining a model that presents a middle-ground between including total-UMIs as a covariate in a GLM and entirely ignoring it in regression. Their proposed model ("regularised NB regression") resembles empirical Bayes but they replace the last (3rd) step of computing the posterior parameter estimate by simply computing residuals which present normalised data, which they are primarily after in this work. The proposed model is intuitive, I would like to see more work on choosing the regularisation, how fast does the proposed model turn to overfit or underfit if the hyper-parameters (number of bins, kernel...) are changed? This is clearly important as in its current form, the hyper-parameters are chosen empirically and their best choice may change as data sets change (e.g. with lower UMI counts per cell or with large number of cells). Secondly, the arguments with respect to parameter estimate uncertainty are weak as they are presented here and should be improved (comment 1). Lastly, simulation benchmarks would benefit the paper and would also strengthen the point that overfitting exists in the model that simply contains the size factor as a covariate (on which the entire paper depends).

Statement on impact:

The impact of overfitting bioligical variation by including total number of UMIs as a regression covariate has not been discussed much in the recent literature and this, and its mitigation as presented here, is a novelty for the field.

Major comments:

- 1: 2.2 Modeling single cell data with a negative binomial distribution leads to overfitting

The authors argue that parameter estimate uncertainty is high based on the distribution of bootstrapped estimators. They identify this large variance in the parameter posterior as overfitting and show that it is reduced if the model is constrained.

-	1.1 Why do you approximate the posterior variance based on boot-strapping rather than using the more common approximation via the fisher information matrix that is also used for Wald tests? It would be good to show this as a reference, too, at least in the supplement, just so that you can convince the reader that these is not an artefact of the bootstrapping procedure.

-	1.2 Clearly, the parameter uncertainty (posterior variance) depends on the number of samples in this scenario. I could not find how many cells were used for the fits presented in Fig. 2. Please add this. Secondly, it would be important to show whether the effect presented in Fig. 2 exists across a range of observation numbers (ie with 100, 1000, 10,000, and 100,000 cells for example).

- 2: 2.3 Applying regularised negative binomial regression for single-cell normalisation, 2.4 Pearson residuals effectively normalise technical differences, while retaining biological variation

-	2.1 I would like to see more work on choosing the regularisation strength here, ie by changing the number of bins used in the kernel regression or bandwidth/kernel choice in the kernel regression.

- 3: 2.5 Downstream analytical tasks are not biased by sequencing depth

-	3.1 You perform differential expression tests based on the residuals, would it not be better to complete the empirical Bayes loop, as I hinted to in the summary, and do differential expression testing directly in the empirical Bayes setting? This seems statistically more coherent.

-	3.2 The discussion on embeddings is nice, could you provide more examples as supplementary figures so that the reader is convinced that no cherry picking took place?

-	3.3 Please clarify log-normalised in the text, I was not sure whether you meant log-transformed or log-transformed and normalised.

- 4: Accuracy of prediction:

-	4.1 You are suggesting a new residual estimation procedure. It would be important to benchmark this on simulated data to show its stability.

-	4.2 You argue that overfitting is an issue in the baseline model of simply including size factors (or number of UMIs etc) as a covariate), it would be good to capture and quantify this issue in terms of mean absolute deviation of the estimate (for example) in simulations. Here, you only argue based on observed posterior variance which would be greatly complemented by simulations which can identify trends in the first moment of the posterior.

**Authors Response**

**Point-by-point responses to the reviewers’ comments:**

We were gratified by the positive comments from the referees. In particular, we were pleased to see that both reviewers appreciated the importance of our proposed statistical model, the regularized negative binomial, to address overfitting of statistical models in single-cell RNA-seq data. Both reviewers also offered helpful and constructive comments. In our revised submission, we introduce new data and computational analyses, and highlight the most important additions below:

•	Application of our method (SCTransform) to datasets where ‘ground truth’ parameters are known. Both reviewers suggested that we apply our method where we had independent knowledge of ground-truth (for example, simulated data or pool-and-split RNA samples). To address this, we include an analysis of data from (Svennson et. al, Nature Methods 2017), where a pool of RNA with known concentration was loaded onto a 10X Genomics run. Since each droplet contains RNA from the same original bulk sample, all variability in the dataset should be technically driven. We show that SCTransform accurately reconstructs robust statistical models that agree with this assumption, while non-regularized models exhibit substantial error due to overfitting..

•	Ensuring robust parameter selection for regularization. Both reviewers requested additional clarification of how our regularization parameter was chosen, and to see if our results were robust to exact parameter choice. To address this, we performed a sensitivity analysis where we varied our regularization parameter choice over an extensive range (0.1 – 100). We justify the default parameter choice we use in the manuscript, and more importantly, show that results are largely unchanged when varying this parameter over a reasonable range that extends an order-of-magnitude, highlighting the robustness of our method.

•	Additional testing and benchmarking of SCTransform. Reviewers asked us to characterize SCTransform performance on additional datasets, listing enteroendocrine cells in the gut, and a continuous differentiation trajectory, as alternative suggestions. We include these analyses in the revised manuscript and reviewer response. In each case, we observe that SCTransform improves upon standard normalization methods or alternative approaches (i.e. scran, Lun et al. [2016b,a]). .

Reviewer 1

In their present manuscript Hafemeister and Satija identify technical variability in single-cell RNA- sequencing data associated with variable total transcript counts across cells as a major confounding factor for the analysis of such data. By stratifying genes into groups of similar abundance they demonstrate that commonly used normalization schemes applying the same size factor to all genes cannot eliminate such kind of variability. To solve this problem for UMI-based count data they propose a gene-specific normalization scheme, utilizing a general linear model with a negative binomial link function, the total UMI count per cell as explanatory variable and the UMI count vector for a given gene as response variable. Since per-gene regression appears to overfit the data, which they conclude from the instability of the parameter estimates during bootstrapping, they propose a regularization scheme. In this approach kernel regression is applied to learn the global trend from genes with similar abundance. The Pearson residuals obtained from these regularized parameters are then used as normalized gene expression estimates. They show that these residuals are indeed not affected by sequencing-depth associated technical noise anymore. Importantly, they demonstrate that, while unconstrained negative binomial regression eliminates biological variability, a Poisson regression is insufficient to remove overdispersed technical noise - in agreement with previous studies. They finally show that their normalization and variance stabilization corrects for sequencing-depth induced batch effects and substantially improves differential gene expression analysis in the presence of such nuisance factors.

The study is technically very well executed and the authors show convincing data demonstrating the power of their approach. The method represents a novel strategy to derive gene-dependent size factors and will be very useful for the community to discover more structure in single-cell datasets but also as a basis for future methods development.

*We thank the reviewer for their generous comments regarding our normalization procedure and statistical modeling of scRNA-seq count data.*

However, I have a few points that need to be addressed:

1)	I like the idea of regularizing the regression parameter. The authors make the point that the parameter instability during bootstrapping is explained by systematic biological variability in the data, which could be different for any subsample of cells. Although this makes intuitive sense, this hypothesis should be directly tested, e.g., by analyzing pool-and-split RNA-samples without any biological variability. For instance, the authors could use the pool-and-split data from Grün et al., 2014, Nature Methods. It would be reassuring if the inferred parameters were more stable for these data.

*This is an excellent point, and we thank the reviewer for suggesting it. To address, we obtained a previously published dataset of bulk human brain RNA that was used as input for a 10X Genomics run Svensson et al. [2017]. Each droplet therefore represents a technical replicate (i.e. the pool-and-split experiment the reviewer suggested). Our findings, shown in Reponse Figure 1 (also in Supp. Figure 3), demonstrate that SCTransform successfully builds robust statistical models for this dataset. In the absence of regularization, we continue to observe substantial variance in the parameter estimates learned independently on each gene. However, due to the nature of the input sample, we know that this cannot reflect biological differences in the noise level of individual genes. Therefore, this analysis conclusively shows that regularization is an essential step for building robust statistical models.*

*Additionally, after applying SCTransform, we examined the residual variance of each gene in the dataset. In contrast to an experiment run on a heterogeneous cell population (where we detect hundreds to thousands of ’variable’ genes), we correctly do not detect a single gene as having high residual variance in this experiment. This demonstrates that SCTransform successfully ascribes all variation in this data to technical, and not biological, sources.*

*We believe this analysis substantially improves the manuscript, and thank the reviewer again for the suggestion.*

*Figure 1: Performing regularized negative binomial regression on a ’Chromium control’ dataset (Svensson et al. [2017]), where each droplet represents a technical replicate of the same human brain bulk RNA pool. A) Top row: Per-gene and regularized parameter estimates for this dataset. Even in the absence of biological variance, there is substantial variation in the estimation of single-gene parameters due to overfitting. Bottom row: For comparison, estimates for the PBMC dataset, randomly downsampled to 2,000 cells. B) Per-gene variance of pearson residuals after applying negative binomial regression. In the control dataset (top), we correctly do not identify genes with high residual variance.*

2)	Another concern is the uncertainty of the regularization for highly expressed genes. This is particularly apparent for the pancreas and the heart dataset in supplementary note 1. The kernel regression for these few highly expressed genes should perhaps incorporate more of the trend from the more lowly expressed genes to obtain a smoother dependency. It is in general not clear to me how the authors motivate their choice for the bandwidth of the kernel regression. This parameter should perhaps depend on the distribution data points (gene density as a function mean expression) which might be different for every dataset.

*We agree with the reviewer that the choice of the kernel bandwidth is important to ensure that we are neither overfitting or under-fitting the data. We also agree that the bandwidth should not be fixed for all datasets, but should depend on the data distribution. In fact, we do select the bandwidth using a data-based selection method by Sheather and Jones [1991], and apologize that this was not clear in the original submission.*

*We do apply an additional scaling factor to the bandwidth to allow for additional smoothing (default value is 3, but can be set by the user). This parameter (bandwidth adjustment factor; BAF), controls the bandwidth between overfitting highly expressed genes (too little smoothing), and obtaining poor fits to highly expressed genes (too much smoothing). The reviewer correctly points out that achieving a reasonable balance is important. We tend to set default BAF values conservatively, ensuring that the trendline reflects a reasonable fit for highly expressed genes, even if there is a slight deviation from the trend for lowly expressed genes. We believe this is conservative because, should we take the opposite strategy, highly expressed genes (including structural and housekeeping genes) can exhibit extremely high values for residual variance, which will negatively affect downstream analyses.*

*To test the robustness of our results to this additional smoothing parameter, we varied BAF over an extensive range (0.1 to 100), and compared the results (Response Figure 2, Supp. Figure 4). Panel A exhibits that values between 1 and 10 all provide reasonable levels of smoothing, while values <0.1 are clearly overfitting the data, and values of >10 are clearly underfitting the data. Our default value (BAF=3) achieves a reasonable balance. More importantly, when observing the output of SCTransform across BAF values of 0.1, 1, 3, and 10, we observe highly concordant results (Panel B, C). To summarize, our bandwidth is automatically set based on the density of the data (as the reviewer suggests), alongside an additional factor that has reasonable default values, and returns robust results across an order of magnitude.*

*Figure 2: Bandwidth adjustment factor (BAF) sensitivity analysis. A) Regularized parameter estimates (red line) for five different values of BAF. Blue points represent single-gene parameter estimates, and are the same in all panels. B) Gene mean versus residual variance relationship for different BAF values. Setting BAF too high fails to correct for the mean/variance relationship in the data. C) Per-gene residual variance for different BAF, compared to the default value (BAF=3). Results for all panels are robust within a range of 1 to 10.*

3)	It was demonstrated in the manuscript that the normalization method successfully eliminates batch effects associated with variability in sequencing depth and improves the identification of differentially expressed genes in the presence of this technical noise. However, the authors did not sufficiently test	in a quantitative manner how their approach affects cell type identification by clustering. The PBMC dataset shown in Figure 6A is not very challenging, due to the limited number of cell types and the clear separation between them. It would be useful to compare clustering after using their method versus log- normalization and other normalization schemes (e.g. scran) for datasets with more cell type variability, ideally comprising sub-types that are more similar to each other. For example, they could analyse the intestinal data from Haber et al., Nature , 2017, and, in particular, test if they can recapitulate the taxonomy

of enteroendocrine cells in Figure 3. This benchmarking should be done in a quantitative way, e.g. ,by computing the Rand index (against a curated annotation from the original manuscript) and the silhouette coefficients.

*We performed the analysis the reviewer suggested, by taking the dataset from Haber et al., Nature , 2017, focusing on the enteroendocrine cells, normalizing the data with three methods (SCTransform (Reg. NB), Log-normalization, and scran Lun et al. [2016b,a]). We agree with the reviewer that this is a valuable benchmark dataset, as the cell type labels have been manually curated and validated, and the dataset was also recently used in Luecken and Theis [2019] to demonstrate best practices for scRNA-seq. That manuscript recommends scran for normalization, but was published before SCTransform was available.*

*Encouragingly, SCTransform exhibited the best performance on this dataset, returning a 40% increase in average silhouette width in comparison to scran (Response Figure 3). However, this is a single dataset with ground truth, and contains only 533 enteroendocrine cells. While we believe that SCTransform performs best-in-class normalization, we believe that there are not a sufficient number of well-annotated ’ground truth’ datasets to perform a conclusive benchmarking analysis using these criteria. Indeed, when breaking these results down by individual cell type (panel B), we see that while SCTransform is the most consistent method across all cell types, there are individual cell types where each of the three methods (including standard log-normalization) achieve high performance. We prefer to be transparent about the challenges in benchmarking, instead of simply highlighting individual results that would appear favorable to our method.*

*However, we do believe that it is important for us to provide quantitative evidence that SCTransform improves upon existing tools. For example, we demonstrated that SCTransform normalization effectively removes technical variation from the data, removing the correlation between sequencing depth and gene expression. In our revised manuscript, we demonstrate that both log-normalization and scran do not effectively remove this relationship (Supplementary Note 1), and that correlation signal remains. This is because scran applies the same normalization strategy to each gene, but as we demonstrate, distinct normalization parameters are required for genes with different abundances. Notably, this limitation is true not only for each of 5 of biological datasets that we explore, but also for the pool-and-split experiment mentioned earlier in point (1) of this response.*

*We conclude that SCTransform effectively normalizes datasets across a wide variety of biological systems and technical controls, and outperforms leading methods in the field.*

4)	Along the same lines the authors should test the effect of their normalization method on trajectory inference. In general, trajectory inference is a challenging task which requires the recovery of continuous manifolds in the data. It would be important to know if the proposed normalization method disrupts continuous trajectories. The authors could apply established methods for trajectory inference (PAGA, diffusion maps, Monocle3 etc.) on systems with well-established lineage information such as the bone marrow after different ways of normalization. Data from Dahlin et al., Blood, 2018 would be an option.

*We agree that effective normalization should maintain the general shape of the manifold, whether discrete or continuous. While there is nothing in the SCTransform method that assumes (or favors) a discrete representation of the single-cell manifold, we agree that its important to ensure that this method does not disrupt continuous trajectories.*

*We considered quantitatively bechmarking SCTransform on a system with multiple developmental branch points, such as the mouse bone marrow. However, we respectfully disagree with the reviewer that the lineage relationships in the bone marrow are well-established, and indeed there is widespread disagreement on the true hematopoietic lineage model. This creates inherent challenges in benchmarking on systems with multiple branchpoints (to our knowledge, there are no datasets of this nature where ground truth is known). However, when we run SCTrans- form on a dataset of the mouse bone marrow from La Manno et al. [2018], we do observe a continuous manifold where the results are strikingly coherent with RNA velocity estimates. This analysis is publicly available on the Seu- rat website http://htmlpreview.github.io/?https://github.com/satijalab/seurat-wrappers/blob/ master/docs/velocity.html, and we reproduce the Figure below (Response Figure 4A). Panel B is the original analysis from the same dataset [La Manno et al., 2018], analyzed with Pagoda, which applies log-normalization. In this case, our method correctly recovers a continuous manifold in the data, while log-normalization does not.*

*Figure 4: Analysis of 2,600 cell dataset of mouse bone marrow progenitor cells. Data was normalized using either SCTransform (panel A), or Pagoda2, which applies log-normalization (panel B). Arrows represent RNA velocity predictions.*

*As a second example, we ran SCTransform on a system with a unidirectional, continuous trajectory, which we have previously shown represents a pseudotemporal and spatial migration of developing inhibitory interneuron progenitors Mayer et al. [2018]. While we cannot have ground truth for the pseudotime of individual cells, we did validate the overall structure of the trajectory with a multitude of in-situ experiments Mayer et al. [2018]. Here, we applied either log-normalization or SCTransform to this dataset, applied diffusion maps to the normalized values, and fit a principal curve through the initial diffusion coordinates in order to estimate a trajectory (Response Figure 5).*

*We obtained strikingly concordant results using both methods. Diffusion maps run on SCTransform-normalized data maintained the continuous structure of the manifold, and the per-cell pseudotime estimates were highly correlated (Pearson correlation = 0.996). Moreover, the dynamics of key regulators was conserved across normalization schemes (genes selected were the same as shown in Fig. 1 of Mayer et al. [2018]). We conclude that SCTransform effectively normalizes data from both discrete and continuous manifolds.*

*Figure 5: Analysis of 21K cell dataset of mouse neuronal progenitor cells from the embryonic ganglionic eminences at day E13.5 (Mayer et al. [2018]). Data was normalized using either SCTransform or log-normalization, revealing nearly identical downstream results. A) Diffusion map coordinates, pink line represents principal curve representing a maturation ’trajectory’. B) Pseudotime (maturation score) estimates are highly concordant between normalization strategies, as are the dynamics of key developmental regulators (panel C)).*

Reviewer 2

The authors set out to establish a normalisation technique for scRNA-seq data that takes the count noise and total number of UMIs into account. They concentrate their efforts on defining a model that presents a middle-ground between including total-UMIs as a covariate in a GLM and entirely ignoring it in regression. Their proposed model ("regularised NB regression") resembles empirical Bayes but they replace the last (3rd) step of computing the posterior parameter estimate by simply computing residuals which present normalised data, which they are primarily after in this work. The proposed model is intuitive, I would like to see more work on choosing the regularisation, how fast does the proposed model turn to overfit or underfit if the hyper-parameters (number of bins, kernel...) are changed? This is clearly important as in its current form, the hyper-parameters are chosen empirically and their best choice may change as data sets change (e.g. with lower UMI counts per cell or with large number of cells). Secondly, the arguments with respect to parameter estimate uncertainty are weak as they are presented here and should be improved (comment 1). Lastly, simulation benchmarks would benefit the paper and would also strengthen the point that overfitting exists in the model that simply contains the size factor as a covariate (on which the entire paper depends).

Statement on impact: The impact of overfitting bioligical variation by including total number of UMIs as a regression covariate has not been discussed much in the recent literature and this, and its mitigation as presented here, is a novelty for the field.

*We thank the reviewer for their kind comments regarding the importance and novelty of our work.*

Major comments:

-	1: 2.2 Modeling single cell data with a negative binomial distribution leads to overfitting The authors argue that parameter estimate uncertainty is high based on the distribution of bootstrapped estimators. They identify this large variance in the parameter posterior as overfitting and show that it is reduced if the model is constrained.

-	1.1 Why do you approximate the posterior variance based on boot-strapping rather than using the more common approximation via the fisher information matrix that is also used for Wald tests? It would be good to show this as a reference, too, at least in the supplement, just so that you can convince the reader that these is not an artefact of the bootstrapping procedure.

*We initially chose to use bootstrapping in the first submission, as this approach can readily be extended to the regularized NB regression for comparison. However, we agree with the reviewer that it is important to examine the standard error (determined by the fisher information matrix), and ensure that the uncertainty estimates are consistent with our bootstrapping estimates. When we consider the parameter uncertainty (as determined from the fisher information matrix) of intercept and slope, and plot this as a function of gene mean, we see a strikingly similar trend compared to the bootstrap approach (see Reponse Figure 6 below, also included as Supp. Figure 2). The higher spread around the trendline for the bootstrapped results is due to the limited number of bootstraps we run. We thank the reviewer for suggesting this, and believe that this new analysis furthers demonstrates the need for regularization to prevent overfitting.*

Figure 6: A) Model parameter standard error as determined by the fisher information matrix (returned from the glm and theta.ml function calls in R). B) Model parameter estimates (black points), trendline for estimates (pink line), trendlines for confidence interval (estimate ± 1.96 times standard error, lightblue lines).

-	1.2 Clearly, the parameter uncertainty (posterior variance) depends on the number of samples in this scenario. I could not find how many cells were used for the fits presented in Fig. 2. Please add this. Secondly, it would be important to show whether the effect presented in Fig. 2 exists across a range of observation numbers (ie with 100, 1000, 10,000, and 100,000 cells for example).

The main dataset has 33,148 cells. We apologize that the reviewer could not find this in Figure 2 (it was stated in the first paragraph of the results section and in the Figure 1 caption, but we have added to the Figure 2 caption as well). We agree that it is an important point to demonstrate the relationship between sample size and parameter uncertainty, and have extended our bootstrap analysis to include downsampled versions of the original dataset. Our downsampling range (1,000 to 32,000) encompasses the sample sizes for the vast majority of current scRNA-seq experiments. As expected, with fewer cells parameter uncertainty increases. However, for the regularized NB model the uncertainty is substantially reduced in comparison to the non-regularized NB. This analysis is shown in the Response Figure 7 below.

*Figure 6: A) Model parameter standard error as determined by the fisher information matrix (returned from the glm and theta.ml function calls in R). B) Model parameter estimates (black points), trendline for estimates (pink line), trendlines for confidence interval (estimate ± 1.96 times standard error, lightblue lines).*

-	1.2 Clearly, the parameter uncertainty (posterior variance) depends on the number of samples in this scenario. I could not find how many cells were used for the fits presented in Fig. 2. Please add this. Secondly, it would be important to show whether the effect presented in Fig. 2 exists across a range of observation numbers (ie with 100, 1000, 10,000, and 100,000 cells for example).

*The main dataset has 33,148 cells. We apologize that the reviewer could not find this in Figure 2 (it was stated in the first paragraph of the results section and in the Figure 1 caption, but we have added to the Figure 2 caption as well). We agree that it is an important point to demonstrate the relationship between sample size and parameter uncertainty, and have extended our bootstrap analysis to include downsampled versions of the original dataset. Our downsampling range (1,000 to 32,000) encompasses the sample sizes for the vast majority of current scRNA-seq experiments. As expected, with fewer cells parameter uncertainty increases. However, for the regularized NB model the uncertainty is substantially reduced in comparison to the non-regularized NB. This analysis is shown in the Response Figure 7 below.*

*Figure 7: Trendlines showing the relationship between sample size (number of cells) and model parameter uncertainty. Parameter uncertainty decreases for more highly abundant genes, or larger dataset sizes. Regularization substantially reduces uncertainty independent of sample size.*

-	2: 2.3 Applying regularised negative binomial regression for single-cell normalisation, 2.4 Pearson residuals effectively normalise technical differences, while retaining biological variation

-	2.1 I would like to see more work on choosing the regularisation strength here, ie by changing the number of bins used in the kernel regression or bandwidth/kernel choice in the kernel regression.

*We agree with the reviewer that the choice of the kernel bandwidth is important to ensure that we are neither overfitting or underfitting the data. We select an initial bandwidth using a data-based selection method by Sheather and Jones [1991]. We next apply an additional factor to the bandwidth to allow for additional smoothing (default value is 3, but can be set by the user). This parameter (bandwidth adjustment factor; BAF), controls the bandwidth between overfitting highly expressed genes (too little smoothing), and obtaining poor fits to highly expressed genes (too much smoothing). The reviewer correctly points out that achieving a reasonable balance is important. We tend to set default BAF values conservatively, ensuring that the trendline reflects a reasonable fit for highly expressed genes, even if there is a slight deviation from the trend for lowly expressed genes. We believe this is conservative because, should we take the opposite strategy, highly expressed genes (including structural and housekeeping genes) can exhibit extremely high values for residual variance, which will negatively affect downstream analyses.*

*To test the robustness of our results to this additional smoothing parameter, we varied BAF over an extensive range (0.1 to 100), and compared the results (Response Figure 2 and Supp. Figure 4). Panel A exhibits that values between 1 and 10 all provide reasonable levels of smoothing, while values <0.1 are clearly overfitting the data, and values of >10 are clearly underfitting the data. Our default value (BAF=3) achieves a reasonable balance. More importantly, when observing the output of SCTransform across for BAF values of 0.1, 1, 3, and 10, we observe highly concordant results (Panel B, C). To summarize, our bandwidth is automatically set based on the density of the data (as the reviewer suggests), alongside an additional factor that has reasonable default values, and returns robust results across an order of magnitude.*

-	3: 2.5 Downstream analytical tasks are not biased by sequencing depth

-	3.1 You perform differential expression tests based on the residuals, would it not be better to complete the empirical Bayes loop, as I hinted to in the summary, and do differential expression testing directly in the empirical Bayes setting? This seems statistically more coherent.

*This is an interesting suggestion. For differential expression, we could indeed formulate the problem as a statistical test where parameter estimates were based on empirical bayes shrinkage. Our use of pearson residuals as intermediate values for differential testing can indeed be thought of as an approximation of this procedure. In particular, an EB framework would allow us to propogate uncertainty from differential expression tests into downstream analyses (for example, network reconstruction, or Gene Set Enrichment Analysis). However, we respectfully suggest that extending the method in this way represents an exciting avenue for future work, and lies beyond the scope of this manuscript.*

-	3.2 The discussion on embeddings is nice, could you provide more examples as supplementary figures so that the reader is convinced that no cherry picking took place?

*We include four additional examples in our revised manuscript and response: an analysis of enteroendocrine cells in the gut (where we examine silhouette coefficients with known ground truth), developing inhibitory interneuron progenitors in the mouse embryo, hematopoietic cells in the mouse bone marrow, and lastly, a control experiment where each ’cell’ represents a technical replicate of a pool of RNA. In our response to Reviewer 1, we discuss the challenges associated with comprehensive benchmarking in the absence of ground truth. However, we consistently show in the manuscript (alongside multiple examples in Supplementary Note 1 that shows all the datasets), that SCTransform effectively normalizes single-cell RNA-seq data by substantially mitigating the relationship between sequencing depth and gene expression levels.*

-	3.3 Please clarify log-normalised in the text, I was not sure whether you meant log-transformed or log-transformed and normalised.

*We agree with the reviewer that this language is not precise and can be misleading. In the revised text we state clearly that standard normalization first reflects a size-factor division (where each gene is scaled by the cell’s total sequencing depth), followed by a log-transformation. We continue to use log-normalization afterwards for brevity, but hope that the clarification is helpful for readers.*

-	4: Accuracy of prediction:

-	4.1 You are suggesting a new residual estimation procedure. It would be important to benchmark this on simulated data to show its stability.

-	4.2 You argue that overfitting is an issue in the baseline model of simply including size factors (or number of UMIs etc) as a covariate), it would be good to capture and quantify this issue in terms of mean absolute deviation of the estimate (for example) in simulations. Here, you only argue based on observed posterior variance which would be greatly complemented by simulations which can identify trends in the first moment of the posterior.

*This is an excellent point, and we thank the reviewer for suggesting it. We were hesitant to generate simulated data, as the results would depend heavily on exactly what model we used to perform simulation, and whether that model agreed with the assumptions of SCTransform. However, we agree that it is important to benchmark the model on a dataset where some ground truth estimates of biological and/or technical noise are available.*

*To address, we obtained a previously published dataset of bulk human brain RNA that was used as input for a 10X Genomics run [Svensson et al., 2017]. Each droplet therefore represents a technical replicate of the same RNA sample (i.e. the pool-and-split experiment suggested by Reviewer 1). Our findings, shown in Response Figure 1 (also as Supp. Figure 3), demonstrate that SCTransform successfully built robust models of this data. In the absence of regularization, we continue to observe substantial variance in the parameter estimates learned independently on each gene. However, due to the nature of the input sample, we know that this cannot reflect biological differences in the noise level of individual genes. Therefore, this analysis conclusively shows that regularization is an essential step for building robust statistical models.*

*Moreover, our regularized parameters reveal a constant relationship between the slope parameter and gene mean (first column of Response Figure 1), a linearly increasing relationship between the intercept parameter and gene mean (second column), and dramatically reduced overdisperson compared to a biological sample (third column). As a consequence of the last result, for this dataset, SCTranform estimates a per-gene variance that is nearly identical to the gene mean (fourth column). In contrast to an experiment run on a heterogeneous cell population (where we detect hundreds to thousands of ’variable’ genes), we correctly do not detect a single gene as having high residual variance in this experiment. All of these results are exactly what is expected from the input data, where there is no genuine biological heterogeneity, and all variation is technically driven.*

*References*

*G. La Manno, R. Soldatov, A. Zeisel, E. Braun, H. Hochgerner, V. Petukhov, K. Lidschreiber, M. E. Kastriti,*

*P. Lönnerberg, A. Furlan, J. Fan, L. E. Borm, Z. Liu, D. van Bruggen, J. Guo, X. He, R. Barker, E. Sundström,*

*G. Castelo-Branco, P. Cramer, I. Adameyko, S. Linnarsson, and P. V. Kharchenko. RNA velocity of single cells.*

*Nature, 560(7719):494–498, 2018. ISSN 14764687. doi: 10.1038/s41586-018-0414-6.*

*M. D. Luecken and F. J. Theis. Current best practices in single-cell RNA-seq analysis: a tutorial. Molecular Systems Biology, 15(6):e8746, jun 2019. ISSN 1744-4292. doi: 10.15252/msb.20188746. URL https://doi.org/10. 15252/msb.20188746.*

*A. T. Lun, K. Bach, and J. C. Marioni. Pooling across cells to normalize single-cell RNA sequencing data with many zero counts. Genome Biology, 17(1):1–14, 2016a. ISSN 1474760X. doi: 10.1186/s13059-016-0947-7. URL http://dx.doi.org/10.1186/s13059-016-0947-7.*

*A. T. Lun, D. J. McCarthy, and J. C. Marioni. A step-by-step workflow for low-level analysis of single-cell RNA-seq data with Bioconductor. F1000Research, 5:2122, 2016b. ISSN 2046-1402. doi: 10.12688/f1000research.9501.2. URL https://f1000research.com/articles/5-2122/v2.*

*C. Mayer, C. Hafemeister, R. C. Bandler, R. Machold, R. Batista Brito, X. Jaglin, K. Allaway, A. Butler,*

*G. Fishell, and R. Satija. Developmental diversification of cortical inhibitory interneurons. Nature, 555: 457, mar	2018. URL	http://dx.doi.org/10.1038/nature25999http://10.0.4.14/nature25999https:*

*//www.nature.com/articles/nature25999{#}supplementary-information.*

*S. J. Sheather and M. C. Jones. A Reliable Data-Based Bandwidth Selection Method for Kernel Density Estimation. Journal of the Royal Statistical Society. Series B (Methodological), 53(3):683–690, 1991. ISSN 00359246. URL http://www.jstor.org/stable/2345597.*

*V. Svensson, K. N. Natarajan, L.-H. Ly, R. J. Miragaia, C. Labalette, I. C. Macaulay, A. Cvejic, and*

*S. A. Teichmann. Power analysis of single-cell RNA-sequencing experiments. Nature Methods, 14:381, mar 2017. URL https://doi.org/10.1038/nmeth.4220http://10.0.4.14/nmeth.4220https://www. nature.com/articles/nmeth.4220{#}supplementary-information*

**Second round of review**

**Reviewer 1**

The authors have done an excellent job in addressing all my previous concerns. The SCTransform method represents a clear improvement over existing normalization methods, and will be a valuable addition to the spectrum of single-cell RNA-seq analysis tools.

**Reviewer 2**

The manuscript has been revised well and now meets my expectations, with a minor exception on the simulation study, see below. I would except such a simulation study to be very little work and the study could be read with suspicion by the community if this was not done. If possible I would recommend to include the changes below in a final revision which I however would not need to see anymore. Overall this is a good manuscript that I expect to be received with much interest by the community.

The choice of split bulk data as validation to avoid simulation is very nice it does however only show "false positive rate control”. It was hence not clear to me that true biological effect parameters other than zero can indeed by recovered by the proposed estimation implementation (here , these confounding effect parameters !=0 would have caused residual variance !=0). Hence I would encourage the authors to include a short simulation study based on model assumptions used in inference with confounding effects such as condition differences.

I would also encourage the authors to include Response Fig. 7 in the manuscript/suppl, very much appreciated. I would also encourage the authors to directly state in the manuscript that differential expression analysis is not only possible based on the residuals but also on the parameter estimates; this would be helpful for, and honest to, readers with a weaker statistics background.

**Authors Response**

**Point-by-point responses to the reviewers’ comments:**

Comment

*Response*
